# The genetic architecture of trait covariation in *Populus euphratica*, a desert tree

**DOI:** 10.3389/fpls.2023.1149879

**Published:** 2023-04-05

**Authors:** Kaiyan Lu, Xueshun Wang, Huiying Gong, Dengcheng Yang, Meixia Ye, Qing Fang, Xiao-Yu Zhang, Rongling Wu

**Affiliations:** ^1^ College of Science, Beijing Forestry University, Beijing, China; ^2^ Department of Artificial Intelligence and Data Science, Guangzhou Xinhua University, Guangzhou, China; ^3^ College of Biological Sciences and Technology, Center for Computational Biology, Beijing Forestry University, Beijing, China; ^4^ Faculty of Science, Yamagata University, Yamagata, Japan; ^5^ Yau Mathematical Sciences Center, Tsinghua University, Beijing, China

**Keywords:** *Populus euphratica*, differential equation, cooperation-competition, QTL mapping, genetic regulatory network

## Abstract

**Introduction:**

The cooperative strategy of phenotypic traits during the growth of plants reflects how plants allocate photosynthesis products, which is the most favorable decision for them to optimize growth, survival, and reproduction response to changing environment. Up to now, we still know little about why plants make such decision from the perspective of biological genetic mechanisms.

**Methods:**

In this study, we construct an analytical mapping framework to explore the genetic mechanism regulating the interaction of two complex traits. The framework describes the dynamic growth of two traits and their interaction as Differential Interaction Regulatory Equations (DIRE), then DIRE is embedded into QTL mapping model to identify the key quantitative trait loci (QTLs) that regulate this interaction and clarify the genetic effect, genetic contribution and genetic network structure of these key QTLs. Computer simulation experiment proves the reliability and practicability of our framework.

**Results:**

In order to verify that our framework is universal and flexible, we applied it to two sets of data from *Populus euphratica*, namely, aboveground stem length - underground taproot length, underground root number - underground root length, which represent relationships of phenotypic traits in two spatial dimensions of plant architecture. The analytical result shows that our model is well applicable to datasets of two dimensions.

**Discussion:**

Our model helps to better illustrate the cooperation-competition patterns between phenotypic traits, and understand the decisions that plants make in a specific environment that are most conducive to their growth from the genetic perspective.

## 1 Introduction

In the growth and development of plants, there are always intimate communication and connection among organs or various parts of the same organ, so that plants can maintain the optimum condition to obtain natural resources and adapt phenotypically to living environment (Niklas and Spatz, 2006; Poorter et al., 2009; Freschet et al., 2015; Weraduwage et al., 2015; Freschet et al., 2018; Tumber-Dávila et al., 2022). The communication and connection are specifically manifested as allocation patterns of biomass (Hermans et al., 2006; Poorter et al., 2012; Wang et al., 2022), which forms different allometric relationships and shapes plant morphology, such as the restrictive relation between aboveground stem and underground roots, height and diameter of stem, etc. (Niklas and Spatz, 2006; Fu et al., 2017). Besides external environmental factors, heredity is a critical driving force for individuals to show a variety of phenotypic characteristics. (Cunniff et al., 2015; Magar et al., 2021).

Quantitative trait locus (QTL) mapping can locate QTLs or genes related to complex quantitative traits on chromosome, which is always the interest of biogenetics and has been successfully applied to many breeding projects (Tang et al., 2010; Wei et al., 2012; Sonah et al., 2015; Fu et al., 2016; Xia et al., 2018). However, traditional QTL mapping mainly focuses on phenotypic data of one trait at one time point, ignoring that plant growth is a dynamic and continuous process. Functional mapping is a QTL mapping method to identify QTLs that regulate the dynamic growth change of target trait during a period of time (Wu and Lin, 2006; Sun and Wu, 2015; Fu et al., 2017).

**Figure 1:**
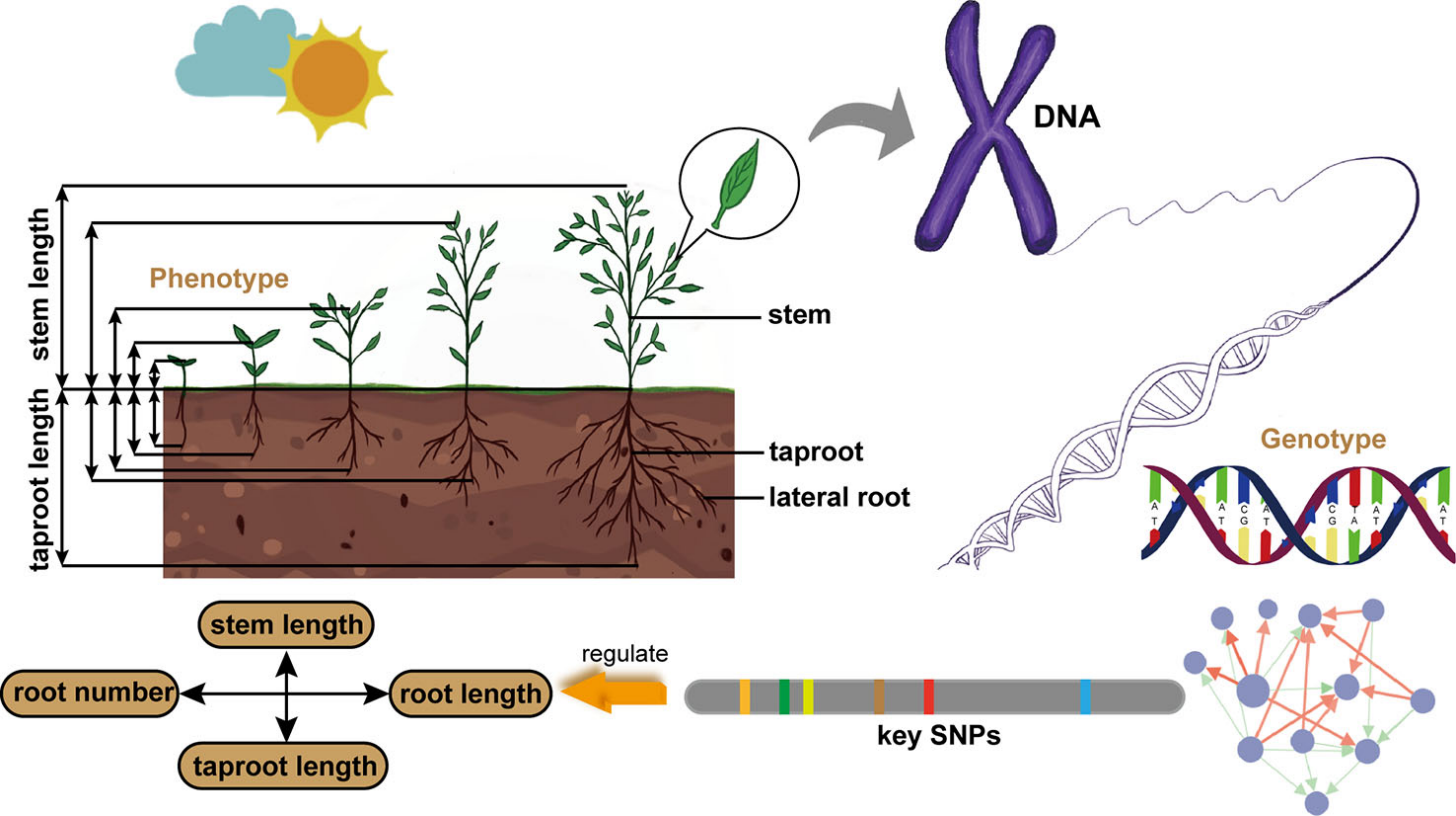
Schematic diagram of our model framework. It is applied to the data of stem length and taproot length from *Populus euphratica* seedlings to identify key QTLs regulating the growth interaction between aboveground and underground, and clarify the architecture of genetic network. It is also used to analyze the data of root number and root length to understand the genetic mechanism.

In this paper, we establish a general and universal model framework, which takes into account the dynamic characteristics of plant growth and the interaction between two traits, screens out key QTLs regulating this pattern, and figures out the genetic structure (**Figure 1**). Our model is based on the Differential Interaction Regulatory Equations (DIRE) to describe the growth of two closely related phenotypic traits and the potential interaction pattern between them. The basic form of this equation comes from the Lotka-Volterra (LV) equation, which described the ecological interaction of two species (Lotka, 1920; Volterra, 1928; May, 1973). Our model identifies a number of genetic loci that determine the cooperation or competition pattern of target traits. According to the genetic effects of these key loci, the genetic network among them is further constructed. We applied the model on two sets of data, stem length - taproot length and root number - root length, which represent the vertical and horizontal relationships of plant architecture, respectively. The balance of competition or cooperation between stem length and taproot length should not only meet the requirements of plants to absorb, synthesize and transport nutrients needed for life, but also ensure plant morphology and stability. Specifically, the extension of root system under the ground makes plants absorb water, minerals elements and other substances in the soil, while stem transports carbohydrate to root and provides support for leaves to ensure effective photosynthesis. And root system shall provide enough resistance to prevent the plant from being pulled out (Modrzyński et al., 2015). The relation between root number and root length reflects the morphological characteristics of root system. Such comparison enables us to have a new understanding of the coordinated variation between traits from two dimensions of plant structure and the genetic mechanism. At the same time, it provides a reference for plant genetic breeding according to the cooperative relationship of traits by means of technical means. Finally, the simulation experiment verifies the reliability of our model.

## 2 Materials and methods

### 2.1 Plant materials

We used the published experimental data to validate the utility of our model (Zhang et al., 2017; Zhang, 2019). A full-sib F1 family of 321 members was derived from hybridization between two dioecious trees of *Populus euphratica* (Zhang et al., 2017). In spring 2014, cut the male and female flowering branches from the two trees and conduct artificial hybridization in water. After more than 4 months, the catkins gradually ripened, and the harvested seeds were cultured in a glass tube (40 mm in diameter and 400 mm in length) which contains 350 ml of 1/2 Murashige and Skoog medium (pH 6.0) under a sterile condition. The tube was placed in a phytotron set at 14-h-day/10-h-night cycle, 28°C day and 22°C night with 800 $\mu mol\;m^{-2}s^{-1}$ photosynthetically active radiation. Two phenotypes, namely stem height (mm) and taproot length (mm) were measured repeatedly for each progeny since seed germination. Measurements were undertaken 16 times: 1, 3, 5, 7, 18, 20, 22, 24 26, 28, 31, 34, 38, 47, 54, 62 days after seed germination, respectively. The full-sib population with the same dioecious parents was planted (Zhang, 2019). In 2014, the flowering branches of the male parent were cultured in water in a phytotron set, and the mature pollen was stored in the EP tube at -20°. The whole plant of the female parent was planted in the greenhouse. After the female flower matures, the male parent's pollen is used for artificial pollination. The mature seeds were obtained in the middle of June. After the seeds were planted in vitro for 4 months, the seedlings were transplanted to the substrate for cultivation, and 408 full-sib offspring were finally obtained. Through the clonal experiment and the expansion and preservation experiment of 408 individuals in the population, 156 groups that can take root on the rooting medium and grow normally into complete plants were finally obtained. Vitro inoculation experiments on 156 groups were conducted. Intercept the terminal buds (about 10 mm, 4-6 leaves) with the same growth status from each single plant and inoculate them into a cylindrical flat-bottomed glass tube (45 mm in diameter and 300 mm in length) for culture. All tubes were added with 260 ml of rooting medium, and placed under the uniform culture conditions in the tissue culture room at 16-h-day/8-h-night cycle, 22°C with 1500lx light. Root length (cm) and root number (count) were collected for each progeny once every 5 days from the 13th day to the 78th day after cultivation. Through the high-throughput sequencing, we obtained 8305 single-nucleotide polymorphisms (SNPs) distributed throughout 19 linkage groups (labeled as Q1-Q8035). All SNP markers can be divided into testcross markers (lm×ll and nn×np) and intercross hybrid markers (hk×hk) according to Mendelian genetic segregation rules, with 6886 and 1419 markers, respectively.

### 2.2 Differential interaction regulatory equations

The biomass allocation among phenotypic traits results in diverse allometric growth relationship. The relationship is a trade-off made by trees after they adapt to the external environment, which largely depends on their internal biological mechanisms. The multiphasic growth model shows that the growth of most organisms is composed of multiple “S”-shaped phases, which is superior to single-phase growth (Koops, 1989; Van der Klein et al., 2020; Gong et al., 2021). The seedling stage of trees is a “S”-shaped phase. According to the basic principles of biophysics and biochemistry, Logistic growth curve shows an "S" shape and consists of exponential growth, linear growth and asymptotic growth (West et al., 2001). Lotka-Volterra equation is derived from the Logistic growth curve and is originally used as dynamic model for growth and decline of amount on predators and prey in biological systems. Its feasibility and effectiveness in describing the microbial abundance under coculture and the predator-prey relationship in the ecosystem have been fully proved (Fujikawa et al., 2014; Jiang et al., 2018). We propose a DIRE model to quantify allometric growth relationship of different traits, and its basic form comes from Lotka-Volterra equation. DIRE describes the cooperation or competition between two traits in the growth process to make full use of survival resources. DIRE model is expressed as


$$\{\begin{array}{c} \frac{dN_{1}}{dt}=D_{1}\left(N_{1}\right)+I_{1}\left(N_{1},N_{2}\right)\\ \frac{dN_{2}}{dt}=D_{2}\left(N_{2}\right)+I_{2}\left(N_{1},N_{2}\right) \end{array}$$

**Figure 2:**
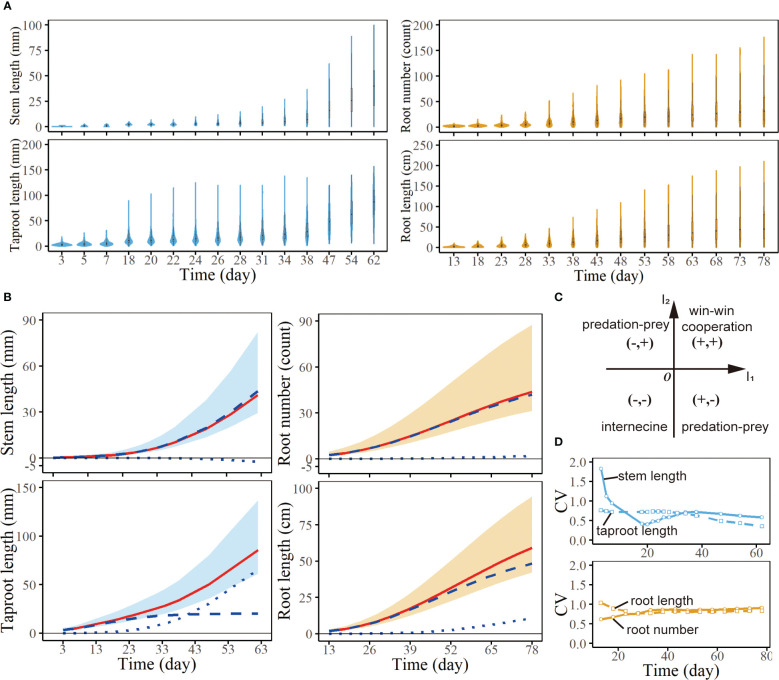
Analysis of the growth of seedlings from a full-sib family of *Populus euphratica*. **(A)** Violin plots of stem length, taproot length, root number and root length of all samples at a series of times. **(B)** Growth curves of stem length, taproot length, root number and root length. Each red line is the fitting curve of average growth for corresponding trait, which consists of independent growth (broke lines) and interactive growth with another trait (dot lines). **(C)** Strategic coordinate system of interaction types between two traits. According to the sign of $\left(I_{1},I_{2}\right)$ , the strategy plane is divided into four regions, representing four interaction types between traits. **(D)** *CV* curves of stem length, taproot length, root number and root length of all samples at a series of times.

DIRE consists of two parts:


$$\{\begin{array}{c} D_{1}\left(N_{1}\right)=r_{1}\cdot N_{1}\cdot (1-{\left(\frac{N_{1}}{K_{1}}\right)}^{s_{1}})\\ D_{2}\left(N_{2}\right)=r_{2}\cdot N_{2}\cdot (1-{\left(\frac{N_{2}}{K_{2}}\right)}^{s_{2}} \end{array}$$

representing the independent growth of target character, and


$$\{\begin{array}{c} I_{1}\left(N_{1},N_{2}\right)=r_{1}\cdot N_{1}\cdot \alpha \cdot \frac{N_{2}}{K_{1}}\\ I_{2}\left(N_{1},N_{2}\right)=r_{2}\cdot N_{2}\cdot \beta \cdot \frac{N_{1}}{K_{2}} \end{array}$$

representing interactive growth of two traits, where $N_{1}$ and $N_{2}$ represent phenotypic values of two characters, $K_{1}$ and $K_{2}$ are the asymptotic value of independent growth, $r_{1}$ and $r_{2}$ represent the independent growth rate, scale parameters $s_{1}$ and $s_{2}$ control the independent growth rate, α and β is the interaction coefficient. The values of α and β reflect interaction types between traits. We can use strategic coordinate system to summarize all interaction types. According to the strategy coordinate system (**Figure 2C**), the strategies for interaction of two traits are divided into three categories: (1) Win-win cooperation: $\left(I_{1},I_{2}\right)\in \left(+,+\right)$ , two traits promote the growth of each other; (2) Predation-prey: $\left(I_{1},I_{2}\right)\in \left(+,-\right)\cup^{}\left(-,+\right)$ , one character is conducive to the other while the latter is harmful to the former. (3) Internecine: $\left(I_{1},I_{2}\right)\in \left(-,-\right)$ , both traits are inhibited by the other.

### 2.3 Statistical model for identifying key QTLs

QTL mapping based on DIRE identifies QTLs that mediate the dynamic growth and interaction between traits (Wu et al., 2011; Sun and Wu, 2015). We design a full-sib mapping population of *n* seedlings. All samples are genotyped for *p* SNPs. Phenotypic values for trait 1 and trait 2 are obtained at a series of time points (*t*=1, …, T), the phenotypic value of seedling *i* is


$${\vec{y}}_{i}=\left({\vec{y}}_{1i};{\vec{y}}_{2i}\right)=\left(y_{1i}\left(1\right),y_{1i}\left(2\right),\ldots ,y_{1i}\left(T\right);y_{2i}\left(1\right),y_{2i}\left(2\right),\ldots ,y_{2i}\left(T\right)\right)$$

where ${\vec{y}}_{i}$ obeys bivariate normal distribution with mean vector $\vec{\mu }$ and covariance matrix Σ , i.e.


$${\vec{y}}_{i}\sim N\left(\vec{\mu },\text{Σ}\right)$$

where the length of $\vec{\mu }$ is 2T, and Σ is a 2T×2T symmetric matrix. In other words, $\vec{\mu }$ and Σ represent the characteristics of the phenotype data for target population. $\vec{\mu }$ is obtained by DIRE, corresponding to parameter set ${\text{Ω}}_{\mu }=\left(K_{1},r_{1},s_{1},\alpha ,K_{2},r_{2},s_{2},\beta \right)$ . Σ can also be estimated by a set of parameters ${\text{Ω}}_{\text{Σ}}$ using first-order structured antedependence (SAD (1)) model (Jaffrézic et al., 2003; Zhao et al., 2005a; Zhao et al., 2005b).

Based on the following hypotheses, we can detect whether a QTL regulates the interaction growth:


$$H_{0}:{\text{Ω}}_{j}=\text{Ω }versus\;H_{1}:{\text{Ω}}_{j}\neq \text{Ω }for\;j=1,\ldots ,J$$

where ${\text{Ω}}_{j}=\left({\text{Ω}}_{\mu_{j}};{\text{Ω}}_{{\text{Σ}}_{j}}\right)$ and $\text{Ω}=\left({\text{Ω}}_{\mu };{\text{Ω}}_{\text{Σ}}\right)$ . The null hypotheses $H_{0}$ means that parameters are independent of genotypes, and the alternative hypotheses $H_{1}$ indicates that there are genotype differences in the parameter sets.

Hypotheses test is realized by maximum likelihood estimation (MLE). The likelihood function of *n* samples for the null hypotheses is


$$L_{0}\left(\vec{y}\right)=\prod_{i=1}^{n}f\left({\vec{y}}_{i};\text{Ω}\right)$$

where $f\left({\vec{y}}_{i}\right)$ is a bivariate probability density function. And the likelihood function for the alternative hypotheses is expressed as


$$L_{1}\left(\vec{y}\right)=\prod_{j=1}^{J}\prod_{i=1}^{n_{j}}f_{j}\left({\vec{y}}_{i};{\text{Ω}}_{j}\right)$$

where *J* is the number of genotypes of target QTL, $n_{j}$ is the number of samples with genotype *j* (j=1,…,J) , which satisfies $\sum_{j=1}^{J}n_{j}=n$ . The likelihood values $L_{0}$ and $L_{1}$ are calculated, respectively, and the test statistic log-likelihood ratio (*LR*) is calculated as


$$LR=-2\left(logL_{0}-logL_{1}\right)$$

*LR* follows the law of chi-square distribution according to its construction principle, and its degree of freedom is the difference between the number of parameters of $H_{0}$ and $H_{1}$ . *p* value is compared with the critical threshold after FDR (False Discovery Rate) correction to reduce false positives.

In the process of hypotheses test solution, parameter estimation is realized by Expectation Maximization (EM) algorithm (Moon, 1996; Do and Batzoglou, 2008). According to EM algorithm, we give an initialization parameter, and estimate the likelihood function value, then iterate and optimize it so that the likelihood function value approximates the local optimal value to obtain new parameters. When the increased value of the likelihood function is less than the target threshold, the iteration ends. We can obtain parameter sets Ω and ${\text{Ω}}_{j}$ of each locus within certain precision, in which the solution of DIRE depends on the fourth-order Runge-Kutta algorithm (Blum, 1962).

### 2.4 Construction of genetic network

The significant QTLs obtained from hypotheses test play key roles in regulating the growth of phenotypic traits. By constructing the genetic network of these QTLs, we can better understand the genetic mechanism of phenotypic variation. We introduce the replication factor equation (Wu and Jiang, 2021), which regards the observed value of the target variable as the sum of its own strategy and interactive strategies with its counterparts. The two parts are referred to as the independent part and the interactive part, respectively, and they are expressed in the form of a nonlinear differential equation system. If these significant QTLs are regarded as a system, the overall genetic effect of each QTL in this system can be decomposed into independent part and the interactive part affected by other QTLs.

$g_{1k}=\left(g_{1k}\left(1\right),\cdots ,g_{1k}\left(T\right)\right)$ 、 $g_{2k}=\left(g_{2k}\left(1\right),\cdots ,g_{2k}\left(T\right)\right)$ denote the overall genetic effects of QTL *k* (*k*=1, 2, …, *K*) for trait 1 and trait 2, respectively. We only retain QTLs that have a greater impact on QTL *k* through variable selection in the LASSO regression model:


$$g_{\cdot k}\left(t\right)=\sum_{k^{\prime }=1,k^{\prime }\neq 1}^{K}b_{k^{\prime }}g_{\cdot k^{\prime }}\left(t\right)+a_{k}+e_{k}\left(t\right)$$

where $g_{\cdot k}$ represents the overall genetic effect of QTL *k*, $b_{k^{\prime }}$ is the regression coefficient of QTL $\;k^{\prime }$ , $a_{k}$ is a constant and $\;e_{k}\left(t\right)$ represents residual. LASSO (Wang and Leng, 2008) selects the most important set of QTLs for the target QTL, and the connections among them are determined by means of a nonlinear differential equation:


$$\frac{dg_{\cdot k}}{dt}=G_{k}^{0}\left(g_{\cdot k};{\text{Θ}}_{k}\right)+\sum_{k^{\prime }=1,k^{\prime }\neq k}^{K^{\prime }}G_{k\leftarrow k^{\prime }}\left(g_{\cdot k^{\prime }};{\text{Θ}}_{k\leftarrow k^{\prime }}\right),k=1,\cdots ,K^{\prime }$$

where the overall genetic effect $g_{\cdot k}$ is decomposed into two parts: $G_{k}^{0}\left(g_{\cdot k};{\text{Θ}}_{k}\right)$ describes the independent genetic effect of QTL *k*; $\sum_{k^{\prime }=1,k^{\prime }\neq k}^{K}G_{l\leftarrow k^{\prime }}\left(g_{\cdot k^{\prime }};{\text{Θ}}_{k\leftarrow k^{\prime }}\right)$ represents interaction produced by other QTLs on QTL *k*, ${\text{Θ}}_{k}$ and ${\text{Θ}}_{k\leftarrow k^{\prime }}$ are parameter sets, respectively. The fourth-order Runge-Kutta algorithm is required to solve the differential equation to determine the extent of interaction among QTLs.

On the other hand, the number of nodes in the network may be more than that of time points. To ensure the accuracy, Legendre orthogonal polynomial (LOP) is firstly applied to fit the genetic effect curve of this QTL and obtain more time point information by interpolation on the curve.

### 2.5 Coefficient of variation

Coefficient of Variation (*CV*) is a normalized measure to evaluate the dispersion of probability distribution. It can be used to compare the dispersion of data among different phenotypic traits on the basis of eliminating influence of dimension. We use it to quantitatively describe the change of dispersion of each phenotypic trait over time. The calculation formula is as follows:


$$CV=\frac{\sigma }{\mu }$$

where σ represents standard deviation and μ represents mean.

### 2.6 Phenotypic variation explained

We define the proportion of phenotypic variation caused by genetic factors (or heritability explained by chosen QTLs) as phenotypic variation explained (PVE), and it is calculated by means of variance analysis. First of all, take the target trait phenotype as the dependent variable and genotypes as the independent variables to conduct generalized linear regression, and then perform variance analysis on the regression result to obtain the PVE value (Xu et al., 2016; Gong et al., 2021).

## 3 Results

### 3.1 Phenotypic variation analysis

Phenotypic traits are expected to be variable among individuals within a species, which is one kind of manifestation of intraspecific variability (Cianciaruso et al., 2009; Albert et al., 2010) and may be caused by genetic factors or phenotypic plasticity induced by environment (Miner et al., 2005). Here, specific performance of this variation is discrepancy in quantitative characters among progeny seedlings, which may not be clear at the seed germination stage, but morphological variation and structural differences within the population become more and more obvious over time. **Figure 2A** illustrates the distribution of stem length - taproot length and root number – root length from F1 population of *Populus euphratica* at a series of time points. Phenotypic values of them both display bigger dispersion with time. The width of the violin gradually narrows, while the vertical height constantly increases, indicating that the differences among progenies increase over time. Taking taproot length as an example for detailed analysis, the height range of the violin at 3 days is 0-20mm, that is, taproot length of all samples does not exceed 20mm at this time. The widest width of the violin shows the concentrated distribution of taproot length in F1 population, and the taproot length of most seedlings is between 1 and 5mm. By the 18th day, the height of the violin has a significant increase, meaning that some samples grow very fast during this period, while some grow slowly. At the moment, the range of the violin is about 1-10mm, i.e., taproot length of most samples is relatively close, about 1-10mm. By the 62nd day, the distribution range of the taproot length has expanded to 0-150mm, and the shape of the violin is very close to a line, indicating that the differences among samples further enlarge, some of the taproots are 150mm long, while others are only about 10mm long. The growth of underground roots also shows similar characteristics, the difference among samples increases with time (**Figure 2A**). This phenomenon is shown on the violin diagram that the violins are both wide and short in the early stage but narrow and high in the late stage. One potential cause of phenotypic variation may be that those samples have different genotypes, suggesting that there may be QTLs regulating woody growth.

The *CV* curve of stem length varies widely, with the maximum value reaching 2 mm at the 3rd day (**Figure 2D**). And it decreases rapidly from the 3rd day to the 18th day, then rises slowly after the 18th day. Above variation trend is caused by the characteristic of sample growth. At the 3rd day, the stem length of most samples is 0, which results in data dispersion. As time goes by, the stems of some samples begin to grow, and the data dispersion begins to decrease. However, there are still some samples with stem length of 0 before the 18th day, so the *CV* values before the 18th day are always large. After 18 days, basically all samples grow stems, and the *CV* value is the lowest in the whole growth process. Then, affected by individual differences, some of the samples grow well, while the others grow slowly, with slight phenotypic changes. Therefore, the degree of data dispersion gradually increases, and the *CV* shows an upward trend. Compared with the stems with slow growth in the early stage, the development of root is earlier, that is, during the growth of seedlings, young individuals tend to allocate more biomass to root growth rather than stem growth. During the whole culture process, the *CV* value of taproot growth remains at about 1, and decreases slightly after 38 days. The *CV* curve of root number rises slowly before the 33rd day and remains basically unchanged after the 33rd day. The *CV* curve of root length decreases slowly before the 33rd day, and remains basically unchanged after the 33rd day, which is completely opposite to that of root number.

### 3.2 Curve fitting and QTL detection of DIRE

Based on the least square method, we used DIRE to fit the growth trajectory of target traits. Unlike some classical growth equations, such as Logistic equation, Gompertz equation and Richards equation, which describe the target character separately, DIRE considers the growth of two traits as a whole, gives the potential internal interaction pattern between them, that is, how trait 1 promotes or inhibits the growth of trait 2, and conversely, how trait 2 affects the growth of trait 1. DIRE can fit the growth data of stem length, taproot length, root number and root length for most progenies well (**Supplementary Figure 1**, **Supplementary Figure 2**, **Supplementary Figure 3** and **Supplementary Figure 4**). The residual distribution diagram shows that the residual value randomly distributed, indicating the robustness of the fitting results (**Supplementary Figure 5**). **Table 1** shows the estimated parameters and evaluation information of the average growth value of the two groups of data by Logistic equation, Gompertz equation and Richards equation and DIRE equation. The evaluation criteria include the residual sum of squares (*RSS*), the coefficient of determination ( $R^{2}$ ), Akaike information criterion (AIC) and Bayesian Information Criterion (BIC). The evaluation result shows that our model has a netter fitting effect than most traditional equations, except that Gompertz equation fits the growth of root number – root length slightly better than DIRE, which indicates that internal interaction indeed affects the growth of the two traits (**Table 1**).

**Table 1:** The estimated parameters and the evaluation information of DIRE.

DIRE				Logistic			
Root number- Root length		Stem length- Taproot length		Root number- Root length		Stem length- Taproot length	
Root number (count)	Root length (cm)	Stem length (mm)	Taproot length (mm)	Root number (count)	Root length (cm)	Stem length (mm)	Taproot length (mm)
57.3800 (*K*_1_)	61.4581 (*K*_2_)	182.1515 (*K*_1_)	20.1145 (*K*_2_)	50.5633 (*A*)	68.6949 (*A*)	71.8931 (*A*)	167.0514 (*A*)
0.1864 (*r*_1_)	0.2565 (*r*_2_)	0.2040 (*r*_1_)	0.1193 (*r*_2_)	0.0695 (*k*)	0.0731 (*k*)	0.0897 (*k*)	0.0574 (*k*)
0.2190 (*s*_1_)	0.1781 (*s*_2_)	0.1694 (*s*_1_)	1.1515 (*s*_2_)	38.3047 (*B*)	53.3358 (*B*)	193.497 (*B*)	33.5669 (*B*)
0.0141 (*α*)	0.0731 (*β*))	–0.0394 (α)	2.3619 (*β*)	/	/	/	/
*RSS* = 2.1483		*RSS* = 5.9005		*RSS* = 10.1548		*RSS* =12.4238	
*R* ^2^ = 0.9997		*R* ^2^ = 0.9995		*R* ^2^ = 0.9987		*R* ^2^ = 0.9990	
*AIC* = –63.0931		*AIC* = 32.8029		*AIC* = –16.3991		*AIC* = –14.4475	
*BIC* = –51.8835		*BIC* = –21.5934		*BIC* = –8.4059		*BIC* = –6.0403	
Gomperz				Richards			
Root number- Root length		Stem length- Taproot length		Root number- Root length		Stem length- Taproot length	
Root number (count)	Root length (cm)	Stem length (mm)	Taproot length (mm)	Root number (count)	Root length (cm)	Stem length (mm)	Taproot length (mm)
67.6613 (*A*)	95.4887 (*A*)	409.2132 (*A*)	797.0535 (*A*)	67.1708 (*A*)	113.8355 (*A*)	113.8355 (*A*)	284.7532 (*A*)
0.0322 (*k*)	0.0325 (*k*)	0.0201 (*k*)	0.0140 (*k*)	0.0313 (*k*)	0.0333 (*k*)	0.0314 (*k*)	0.0208 (*k*)
5.3221 (*B*)	5.9905 (*B*)	7.9497 (*B*)	5.3180 (*B*)	0.2922 (*B*)	0.245 (*B*)	0.5157 (*B*)	0.1581 (*B*)
/	/	/	/	0.9402 (*m*)	0.9700 (*m*)	0.9008 (*m*)	0.9637 (*m*)
*RSS* = 1.9778		*RSS* = 8.9263		*RSS* = 4.64788		*RSS* = 37.4267	
*R* ^2^ = 0.9998		*R* ^2^ = 0.9993		*R* ^2^ = 0.9998		*R* ^2^ = 0.9998	
*AIC* = –62.2073		*AIC* = –24.3657		*AIC* = –34.2822		*AIC* = 24.1250	
*BIC* = –54.2141		*BIC* = –15.9586		*BIC* = –23.6246		*BIC* = 34.7827	

Our model divides the overall growth of the target trait into two parts, namely, the independent growth part and the interaction part. According to the symbols of $\left(I_{1},I_{2}\right)$ , which are determined by the symbols of interaction parameters α and β , we can judge the interaction strategies between two traits (**Table 1**; **Figure 2C**). The interaction strategy of stem and taproot belongs to predation-prey strategy. Stem length growth is inhibited by taproot length growth; conversely, taproot growth benefits from stem length (**Figure 2B**). The growth pattern of root number and root length follows a win-win cooperation strategy, they promote the growth of each other. However, the degree of mutual benefit is not completely equal. It is obvious that the interactive curve of root length is significantly higher than 0 horizontal line, while that of root number is close to 0 horizontal line, meaning that root length growth gains more benefits from root number growth (**Figure 2B**).

**Figure 3:**
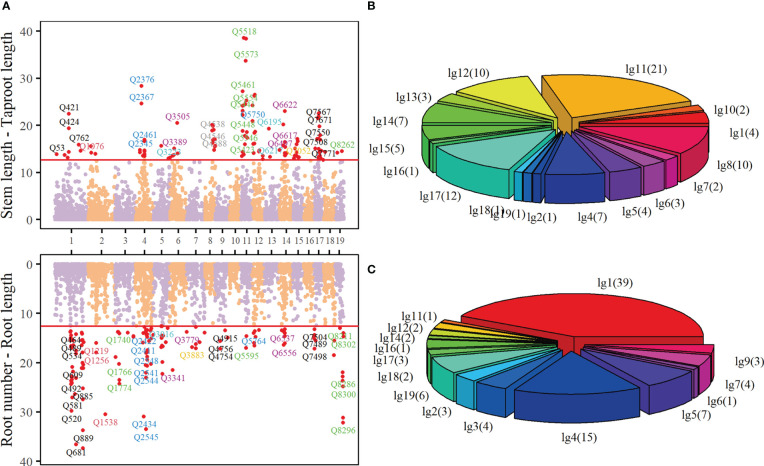
Manhattan plot **(A)** of p-values over 19 linkage groups of *Populus euphratica*, where the threshold (red horizontal line) is determined by FDR correction. The proportion of significant QTL regulating stem length - taproot length **(B)** and root number - root length **(C)** in 19 linkage groups.

DIRE quantitatively describes the growth of two characters as a whole interacting with each other. Different growth patterns show different growth curve trajectories, corresponding to different parameter sets ${\text{Ω}}_{\mu }=\left(K_{1},r_{1},s_{1},\alpha ,K_{2},r_{2},s_{2},\beta \right)$ . Significant QTLs that regulate the growth of target traits can be identified from the whole genome through a series of hypothesis tests on parameter sets. We obtained 94 significant QTLs regulating the growth of stem length - taproot length and 93 significant QTLs regulating the growth of root number - root length at the threshold of $0.1\times 10^{-10}$ (**Figure 3A**). These QTLs are sporadically distributed in each linkage group. QTLs regulating the growth of stem length - taproot length mainly locate in Linkage Group 8, 11, 12 and 17, accounting for 10.64%, 22.34%, 10.64% and 12.77% of all key QTLs, respectively (**Figure 3B**).

A number of QTLs are annotated to biological functions closely related to the growth of *Populus euphratica*, or are homologous with some genes that play key roles in the growth of other trees. For example, the gene annotated by Q3009 (nn_np_12066, Linkage Group 5) encodes pentapeptide repeat containing protein At4g14850, which is a homologous protein of LOI1 (Zhang et al., 2017). LOI1 is a component of mitochondria and a regulator of isoprenoid biosynthesis, affecting the synthesis of ATPQ, RNA and other biological macromolecules (Kobayashi et al., 2007). LOI1 interacts with MEF14 and participates in mitochondrial editing and cytochrome c, while the cytochrome c, as an important component of the electron transport chain, has the oxidation-reduction ability, participates in the oxidative phosphorylation KEGG pathway, and is active in mitochondria, organelle envelope and cytoplasm. Q2345(nn_np_11278, Linkage Group 4) is homologous with LOC7486368, the gene encoding sugar transport protein (STP) 10 in *Poplus tricocarpa*. STP is a plant-specific transport protein, which is responsible for absorbing glucose from the apoplast into plant cells. It is an important member of monosaccharide transporters and plays a crucial role in the division and morphogenesis of organs such as seeds. They are the components of organ development in co-plastic isolated tissues, such as seeds, pollen and fruit. (Cheng et al., 2015; Rottmann et al., 2018; Bavnhøj et al., 2021). The key QTLs that control the growth of root number - root length are mainly distributed in Linkage Groups 1 and 4, in which Linkage Groups 1 contains the largest number of QTLs, reaching 39, accounting for 41.94% of all the key QTLs (**Figure 3C**). These QTLs are also annotated to rich biological functions. In particular, some QTLs are closely related to the growth of plant roots. The gene annotated by Q910 (nn_np_11836) in Linkage Group 1 encodes a leucine-rich repetitive receptor-like protein kinase family protein, which is mainly located in the membrane system. This gene is involved in ATP synthesis, cytokinin regulation and endocytosis transport of cell membrane. In Arabidopsis research, it has been proved that this gene controls root growth by mediating cytokinin. The increase of At2g33170 will promote the growth of root length in Arabidopsis (Hove et al., 2011). **Supplementary Table S1** and **Supplementary Table S2** show the basic information of these key QTLs, including the linkage group, genetic distance, annotated gene function information, etc.

### 3.3 Genetic architecture analysis

**Figure 4:**
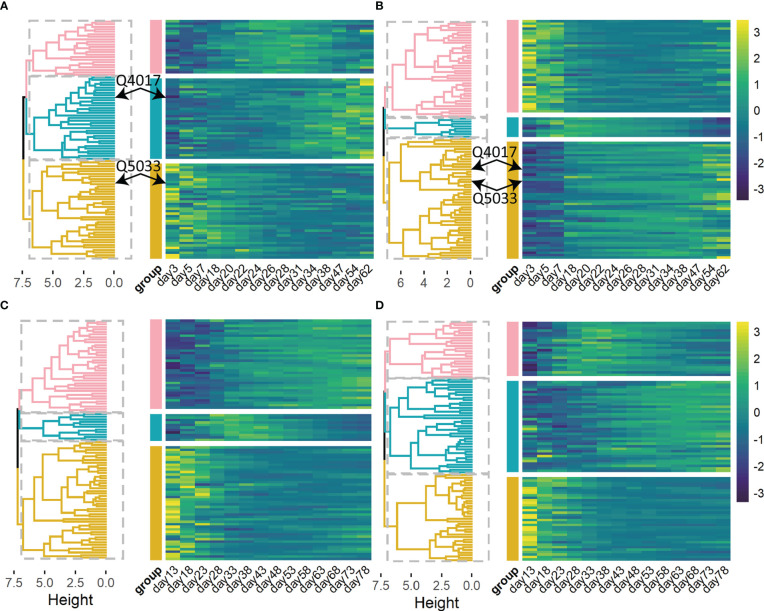
Heat maps and hierarchical clustering trees of heritability are explained by 93 significant QTLs for stem length **(A)**, taproot length **(B)**, and 94 significant QTLs for root number **(C)**, root length **(D)** of *Populus euphratica*, respectively. Heat maps of heritability are explained by several selected QTLs. Significant QTLs are clustered into 3 categories (pink, green and yellow).

Genetic factors contribute to phenotypic variation. We calculated heritability i.e. phenotypic variation explained (PVE) of 93 and 94 significant QTLs by quantifying the dynamic genetic contribution of markers to growth (**Figure 4**). There is dramatic variation in the temporal pattern of heritability (PVE) for stem length, and these patterns can be roughly clustered into three categories (**Figure 4A**): ① PVE increases and then decreases (pink); ② PVE increases monotonously over time (yellow); ③ PVE decreases consistently over time (green). And the temporal patterns of heritability for taproot length can be sorted into similar three groups (**Figure 4B**). Among the significant loci, the variation trends of PVE at the same QTL for two traits are different and even completely opposite. Taking Q5033 as an example, for taproot length, PVE of it increases monotonously over time, while PVE of it for the growth of stem length decreases with time, displaying a completely opposite trend. It means that the genetic contribution to the growth of taproot length increases with time, and the genetic contribution to the growth of stem length decreases gradually. At the same time, the PVE variation trend of a pair of characters on the same QTL may be similar, for example, PVE of Q4017 for stem length and taproot length (**Figures 4A, B**) both increases with time. The temporal patterns of heritability for other traits (**Figures 4C, D**) can also be sorted into three clusters.

**Figure 5:**
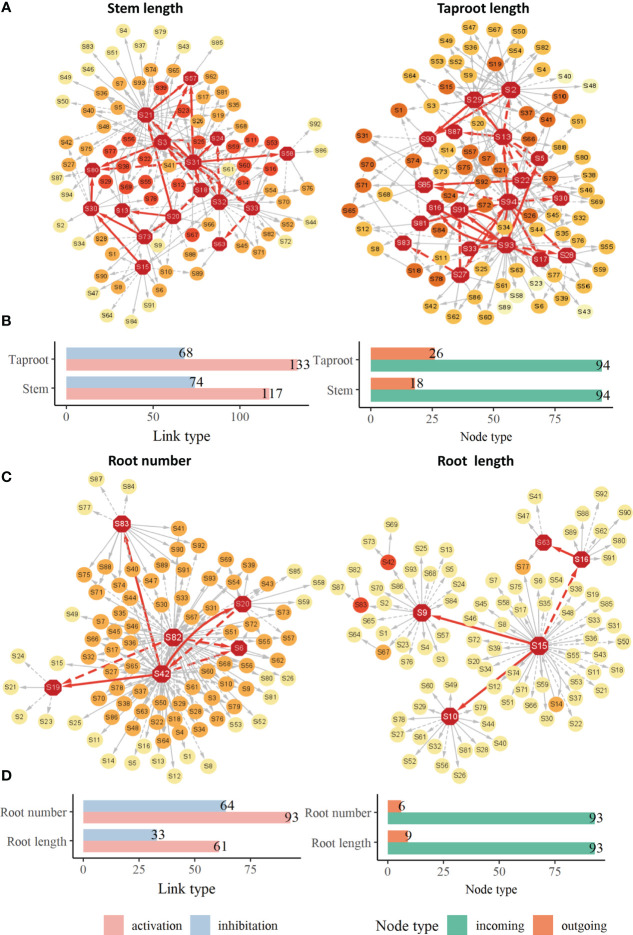
Genetic effect networks **(A)**, bar chart for activating links and inhibitory links and bar chart for outgoing nodes and incoming nodes in networks **(B)** for stem length and taproot length. Genetic effect networks **(C)**, bar chart for activating links and inhibitory links and bar chart for outgoing nodes and incoming nodes in networks **(D)** for root number and root length. Each node represents a QTL. The edges connecting nodes represent the interaction among QTLs, the dotted links represent the inhibitory effect, the solid links represent facilitation. The red nodes represent QTLs that stimulate or inhibit other QTLs.

The growth of phenotypic traits is largely influenced by key QTLs. Although these identified QTLs jointly regulate the overall growth of target trait, there are great differences in their roles and importance in the genetic network. These QTLs do not exist independently, they are subject to epistatic effects from other QTLs besides direct genetic effects produced by themselves. We renumbered these QTLs and constructed their genetic effect network to clarify their interactions and how these QTLs directly or indirectly affect the growth of target traits. These genetic networks, including stem length network, taproot length network, root number network and root length network, share similarities in structure (**Figure 5**). Several dominant core QTLs regulate most other QTLs, while most QTLs are in a relatively secondary position, they receive epistatic influence from core QTLs in the form of stimulation or inhibition. For example, in stem length network (**Figures 5A, B**), only 28 core QTLs play major regulatory roles, accounting for 29.8% of all nodes. In the root number network (**Figures 5C, D**), only 6 QTLs play regulatory roles, accounting for only 6.45% of all QTLs, in which the roles of S42 (Q1538, hk_hk_1218) and S82 (Q7296, nn_np_9326) are particularly critical. The number of links generated by the two QTLs accounts for 79.6% of the total network links, and there are also links among them, which means that the leading QTLs not only produce direct genetic effects, but also indirectly affect the expression of other QTLs, thus directly and indirectly affect the growth of root. In the genetic network of other traits, the situation is similar. On the other hand, genetic networks contain a variety of mutual regulatory relationships between QTLs, including unidirectional and bidirectional relationships through promotion or inhibition, which are determined by the characteristics of genetic effects produced by the QTL itself and the QTL interacting with it. In the taproot network (**Figure 5A**), there are mutually promotion QTL pairs S16↔S93. In the genetic network of root number (**Figure 5C**), there is S42↔S20, in which S42 stimulate S20, while S20 inhibits the expression of S42. By counting the number of link type between QTLs in each genetic network, **Figure 5B** and **Figure 5D** shows that the number of activation links is more than the number of inhibition links, and the former is 1-2 times that of the latter.

### 3.4 Simulation

In order to further verify the accuracy and validity of our model, we simulated and analyzed the growth data of *Populus euphratica* under different sample size (*n*) and heritability ( $H^{2}$ ). The sample size *n* is set to 100 and 300 respectively, and the heritability $H^{2}$ , that is, the proportion of genetic variation in the simulated phenotypic variation, is set to 0.05 and 0.1 respectively. According to the analysis results on real data, the growth of stem length and taproot length is regulated by Q681 of linkage group 1, and the growth of root number and root length is regulated by Q5518 of linkage group 11. In the simulation experiment, we conducted 100 effective simulation calculations based on the parameters of the two genotypes of above two significant QTLs under different sample sizes and heritability. Since Q681 and Q5518 are markers of test cross and have two genotypes, we record these two genotypes as *ll* and *lm* respectively. Our model is applied to analyze phenotypic data and genotype data obtained from simulation experiment. The Maximum Likelihood Estimation algorithm is implemented to obtain the likelihood function values of the null hypothesis and alternative hypothesis in hypothesis testing and parameter set ${\text{Ω}}_{\mu }=\left(K_{1},r_{1},s_{1},\alpha ,K_{2},r_{2},s_{2},\beta \right)$ combined with fourth-order Runge-Kutta, Expectation Maximization (EM), Least Square Method and local search optimization algorithm BFGS. We can find that there are some differences between the parameters obtained from the simulation experiment and the real parameters (**Tables 2**, **3**), but the differences come smaller with the increase of heritability or sample size. Here, the parameter values obtained from the simulation experiment are the average values of 100 groups of parameters.

**Table 2:** Comparison between the real parameter set and the parameter set of simulation experiment results for stem length **(mm)** and taproot length **(mm)**.

	TRUE		*H* ^2^ = 0.05				*H* ^2^ = 0.1			
			*n*=100		*n*=300		*n*=100		*n*=300	
	*lm*	*ll*	*lm*	*ll*	*lm*	*ll*	*lm*	*ll*	*lm*	*ll*
*K*_1_	50.7415	58.6190	53.3361	52.9417	54.2914	58.1174	52.9674	58.7834	53.7712	56.8819
*r*_1_	0.1473	0.1444	0.1240	0.1404	0.1230	0.1402	0.1409	0.1406	0.1452	0.1411
*s*_1_	0.2641	0.3651	0.3785	0.4437	0.3418	0.3976	0.3113	0.3928	0.2713	0.3865
α	-0.0024	0.0618	-0.0153	0.1162	-0.0156	0.0838	-0.0179	0.0699	-0.0121	0.0718
*K*_2_	62.1173	68.2784	58.7068	70.1069	66.0069	69.8684	62.8763	70.2909	58.2741	67.4328
*r*_2_	0.2530	0.1893	0.1598	0.1912	0.1504	0.1772	0.1991	0.1869	0.2084	0.1868
*s*_2_	0.1664	0.2843	0.3349	0.2930	0.3053	0.3040	0.2359	0.2909	0.2213	0.2944
β	0.0315	0.1340	0.0809	0.1364	0.0086	0.1375	0.0266	0.1311	0.0579	0.1421

**Table 3:** Comparison between the real parameter set and the parameter set of simulation experiment results for root number **(count)** and root length **(cm)**.

	TRUE		*H* ^2^ = 0.05				*H* ^2^ = 0.1			
			n=100		n=300		n=100		n=300	
	*lm*	*ll*	*lm*	*ll*	*lm*	*ll*	*lm*	*ll*	*lm*	*ll*
*K*_1_	188.4385	193.0498	78.7291	158.5078	157.5352	165.9129	157.5352	165.9129	155.3674	164.7949
*r*_1_	0.1179	0.1288	0.0934	0.1055	0.1057	0.1141	0.1056	0.1141	0.1049	0.1143
*s*_1_	0.6105	0.4320	1.1293	0.7173	0.9500	0.6104	0.9500	0.6104	0.9846	0.5930
α	-0.789	-0.3113	-0.2289	-0.3624	-0.8135	-0.3354	-0.8135	-0.3354	-0.8372	-0.3287
*K*_2_	23.2003	26.3170	24.7709	26.3031	22.9606	26.2959	22.9606	26.2959	22.9184	26.4386
*r*_2_	0.0978	0.0976	0.0844	0.0892	0.0916	0.0930	0.0916	0.0930	0.0915	0.0930
*s*_2_	1.2007	1.2964	1.2145	1.3195	1.2148	1.3108	1.2148	1.3108	1.2191	1.3151
β	2.4344	2.4917	2.4205	2.5593	2.4739	2.5427	2.4739	2.5427	2.4979	2.5627

**Figure 6:**
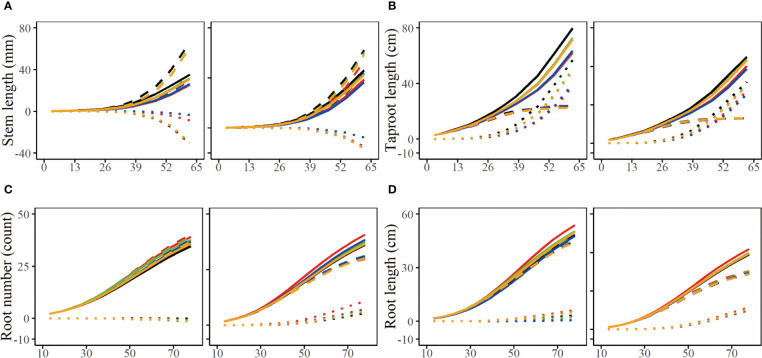
Growth curves of stem length **(A)**, taproot length **(B)**, root number **(C)** and root length **(D)** of two genotypes with a heritability of 0.05 and 0.1. The overall growth (solid line) for each trait is decomposed into independent part (broken line) and interactive part (dotted line). The sample sizes are 100 and 300. Black line represents the real curve, red represents the estimation curve with the sample size of 100 and a heritability of 0.05, blue represents the estimation curve with the sample size of 100 and a heritability of 0.1, the green represents the estimation curve with the sample size of 300 and a heritability of 0.05, the orange represents the estimation curve with the sample size of 300 and a heritability of 0.1.

The parameter set of the simulation result is presented in the form of growth curve, which helps us more intuitively observe the similarity and difference between the simulation experiment result and the real growth data (**Figure 6**). According to the average parameter values of estimated parameters of 100 groups of simulation experiments under four simulation conditions respectively (*n*=100, 300; $H^{2}$ =0.05, 0.1), we depicted their corresponding growth curves, including the overall growth curve, independent growth curve and interactive growth curve. By comparing the estimation curves of different heritability levels and different simulated sample sizes with real growth curves, we find that the simulation effect of the estimation curve with heritability of 0.1 is better than that of 0.05, and the simulation effect of the estimation curve with simulation quantity of 300 is obviously better than that of 100. In **Figure 6**, the simulation curve with larger heritability or sample size is closer to the real curve, especially the curve with heritability of 0.1 and sample size of 300, which is the closest to the real curve. This is consistent with our expected results, that is, the simulation effect increases with the increase of sample size and heritability.

## 4 Discussion

In the process of tree growth and development, different organs or different tissues of the same organ do not exist independently. We cannot ignore the interaction between them. DIRE not only quantitatively describes cooperation and confrontation between two traits under a certain environment, but also explains the genetic regulation mechanism behind this collaboration. It is calculated on two groups of data with different characteristics: stem length - taproot length, root number - root length. Simulation experiments verify that all groups of data have achieved good results, indicating the validity and universality of our model. The characters that affect and restrict each other in the growth process of tree include but are not limited to above traits. With appropriate modifications and adjustments, DIRE can be extended to describe the relationship between more characters such as height and diameter of stem, stem and leaves, then tap the genetic driving force behind them.

The competition and cooperation between different characters exist in the whole process of tree growth. In this paper, we mainly focus on the seedling stage, which is only a very short period for trees whose life span in years, and its time units are days. But this does not mean that our model is only suitable for describing the growth of the seedling stage. We can extend it to describe the dynamic changes of characters in the process of tree growth for several years or even more than ten years, or consider different growth stages of trees.

The data materials in this paper are cultivated under the condition of controlling environmental variables, but in reality, the environment in which trees grow is uncontrollable, and the genetic effect in the whole growth and development of trees is also affected by environmental stimulus. Therefore, our model can be applied to the response of the genetic structure of tree growth to environmental changes, including in different light, temperature, soil and so on (Srikanth and Schmid, 2011; Xie et al., 2012; Boyce et al., 2020). In particular, by comparing the genetic regulation mechanism of tree growth under some extreme conditions, such as drought, salt stress, etc., it can help to explore the survival mechanism of plants under extreme conditions from a genetic perspective (Wang et al., 2021).

In addition, the model can be extended from two-dimensional to multi-dimensional model, so as to mine the key quantitative trait loci that regulate the synergetic growth of multiple complex traits. It is more reasonable to consider the integrity of plant growth, but this expansion will largely increase the complexity of the model and computational difficulty to a certain extent.

We have carried out simulation experiments based on real data, and the simulation results show that our model has good statistical properties, which provides an effective analytical framework for describing the dynamic interaction patterns between different traits during plant growth and development.

## Data availability statement

The datasets presented in this study can be found in online repositories. The names of the repository/repositories and accession number(s) can be found below: https://github.com/Lukaiyan/2datasets, github.

## Author contributions

KL performed data analysis and wrote the manuscript. X-YZ and RW conceived of the idea, designed the model and contributed to the revision of the manuscript. XW participated in the design of the data analysis. HG and DY participated in data analysis and gene annotation. MY provided data of *Populus euphratica*, interpretation of data and guidance for data analysis. QF provides guidance for data analysis and simulation. All authors contributed to the article and approved the submitted version.
