# Correction: The genetic architecture of trait covariation in *Populus euphratica*, a desert tree

**DOI:** 10.3389/fpls.2026.2014733

**Published:** 2026-10-08

**Authors:** Kaiyan Lu, Xueshun Wang, Huiying Gong, Dengcheng Yang, Meixia Ye, Qing Fang, Xiao-Yu Zhang, Rongling Wu

**Affiliations:** 1 College of Science, Beijing Forestry University, Beijing, China; 2 Department of Artificial Intelligence and Data Science, Guangzhou Xinhua University, Guangzhou, China; 3 College of Biological Sciences and Technology, Center for Computational Biology, Beijing Forestry University, Beijing, China; 4 Faculty of Science, Yamagata University, Yamagata, Japan; 5 Yau Mathematical Sciences Center, Tsinghua University, Beijing, China

**Keywords:** *Populus euphratica*, differential equation, cooperation-competition, QTL mapping, genetic regulatory network

The funder “National Key Research and Development Program of China, No. 2021YFB2301601” was erroneously included.

The original version of this article has been updated.

