# Supplementary material for: The genetic architecture of trait covariation in Populus euphratica, a desert tree
Source: Front Plant Sci. 2023 Apr 5;14:1149879. doi: 10.3389/fpls.2023.1149879 (PMC10113509; doi:10.3389/fpls.2023.1149879)
Supplement: Supplementary file 1 [file DataSheet_1.docx]

**Supplementary Figure 1**


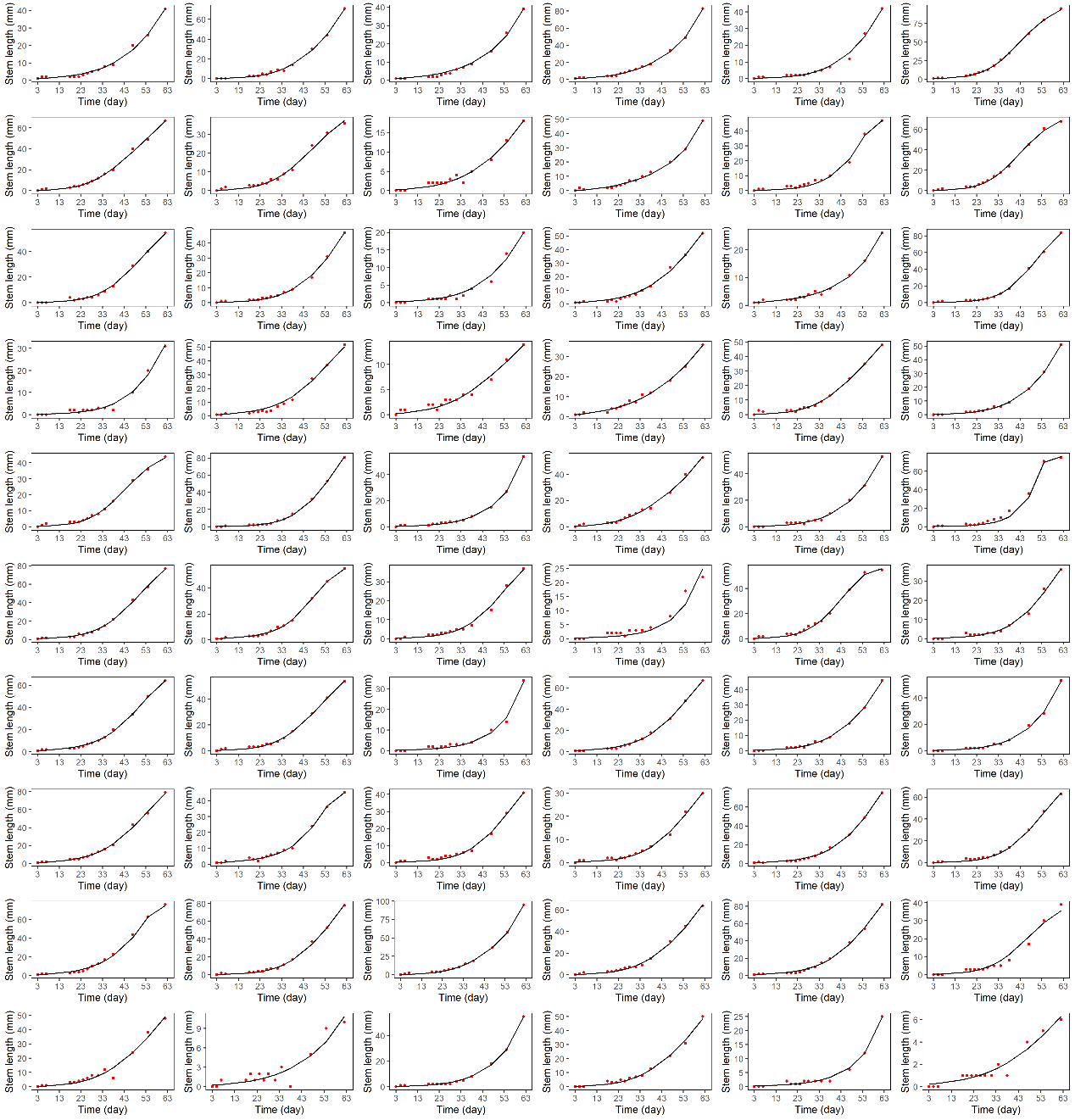


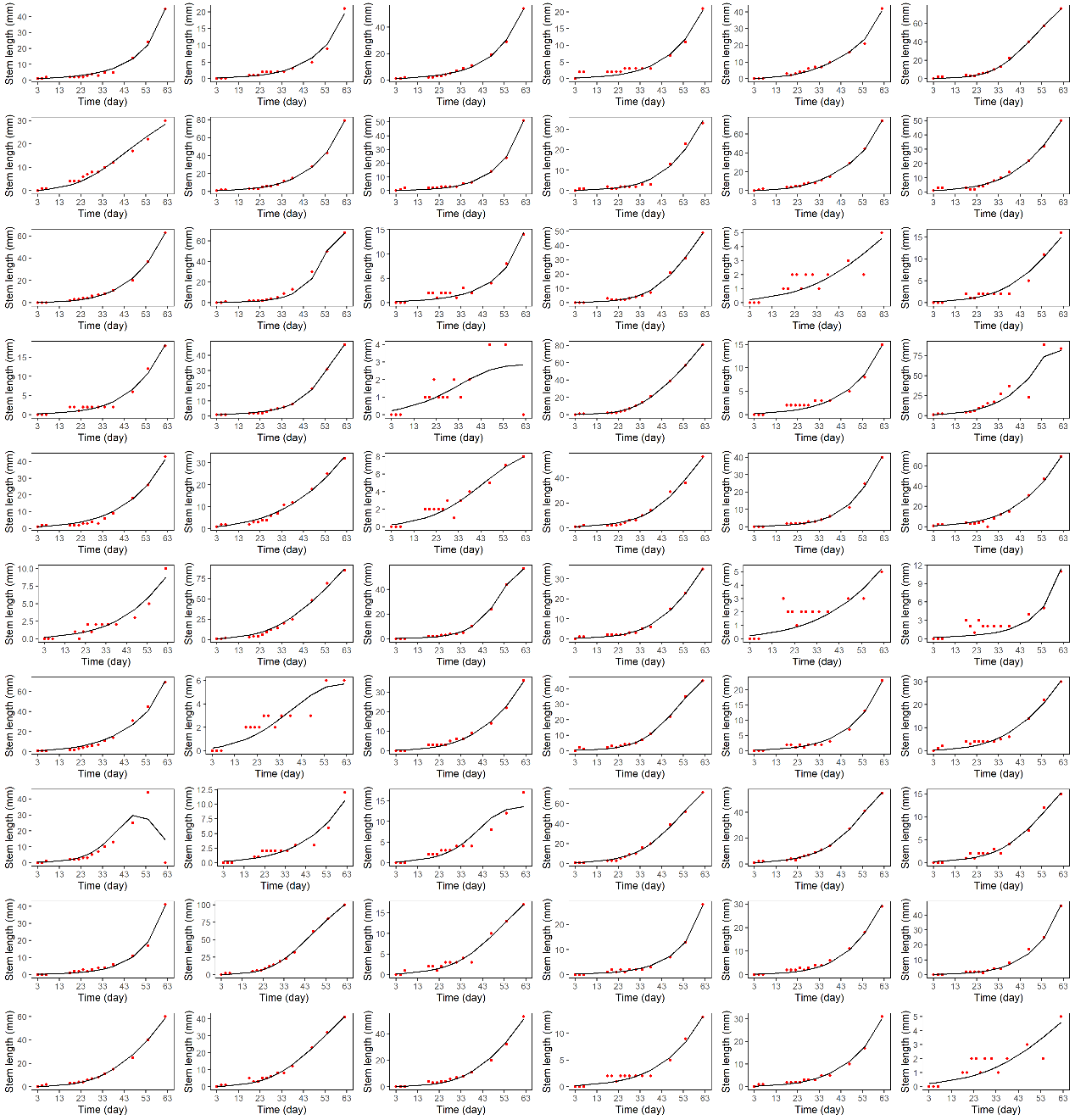


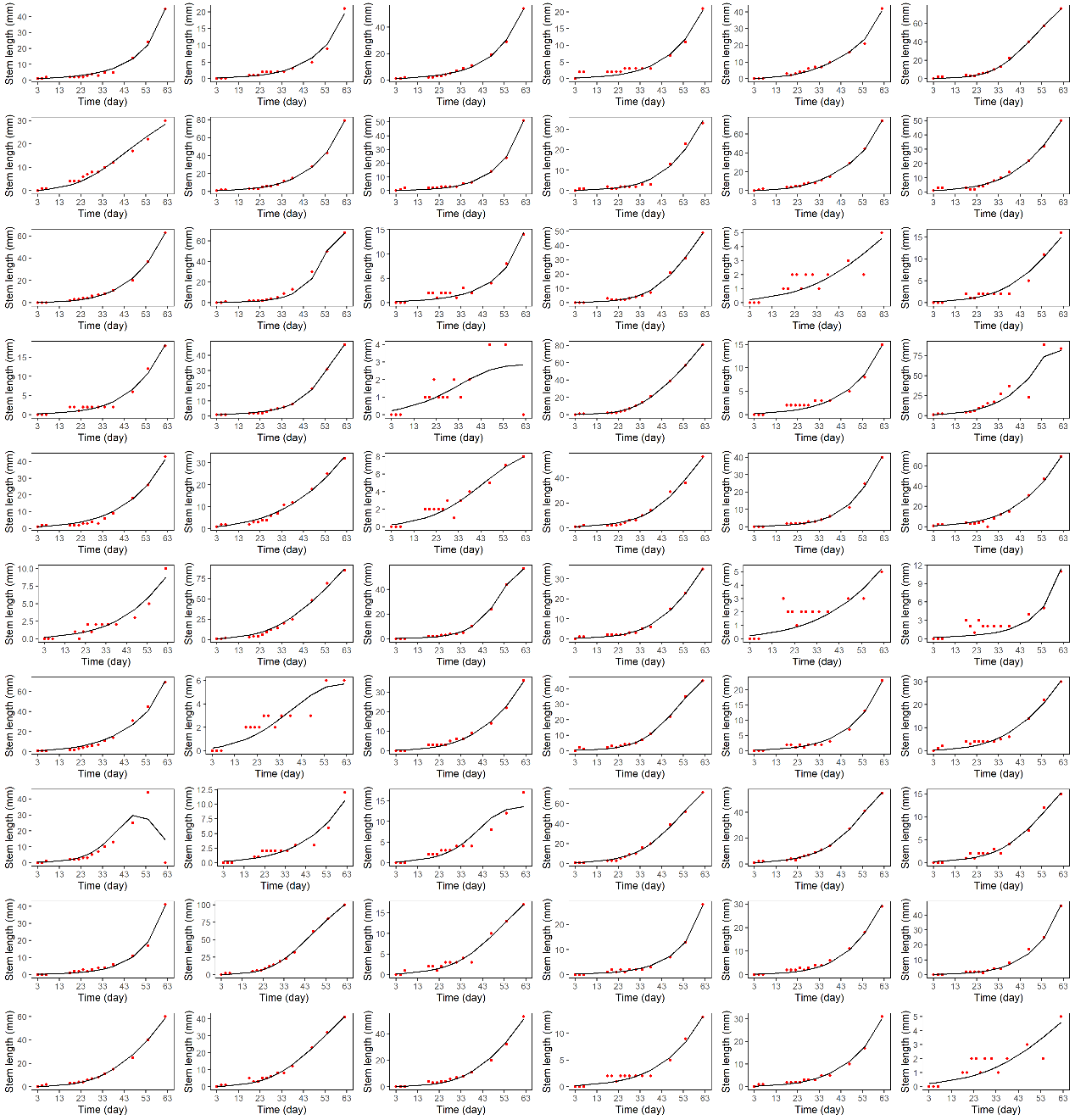


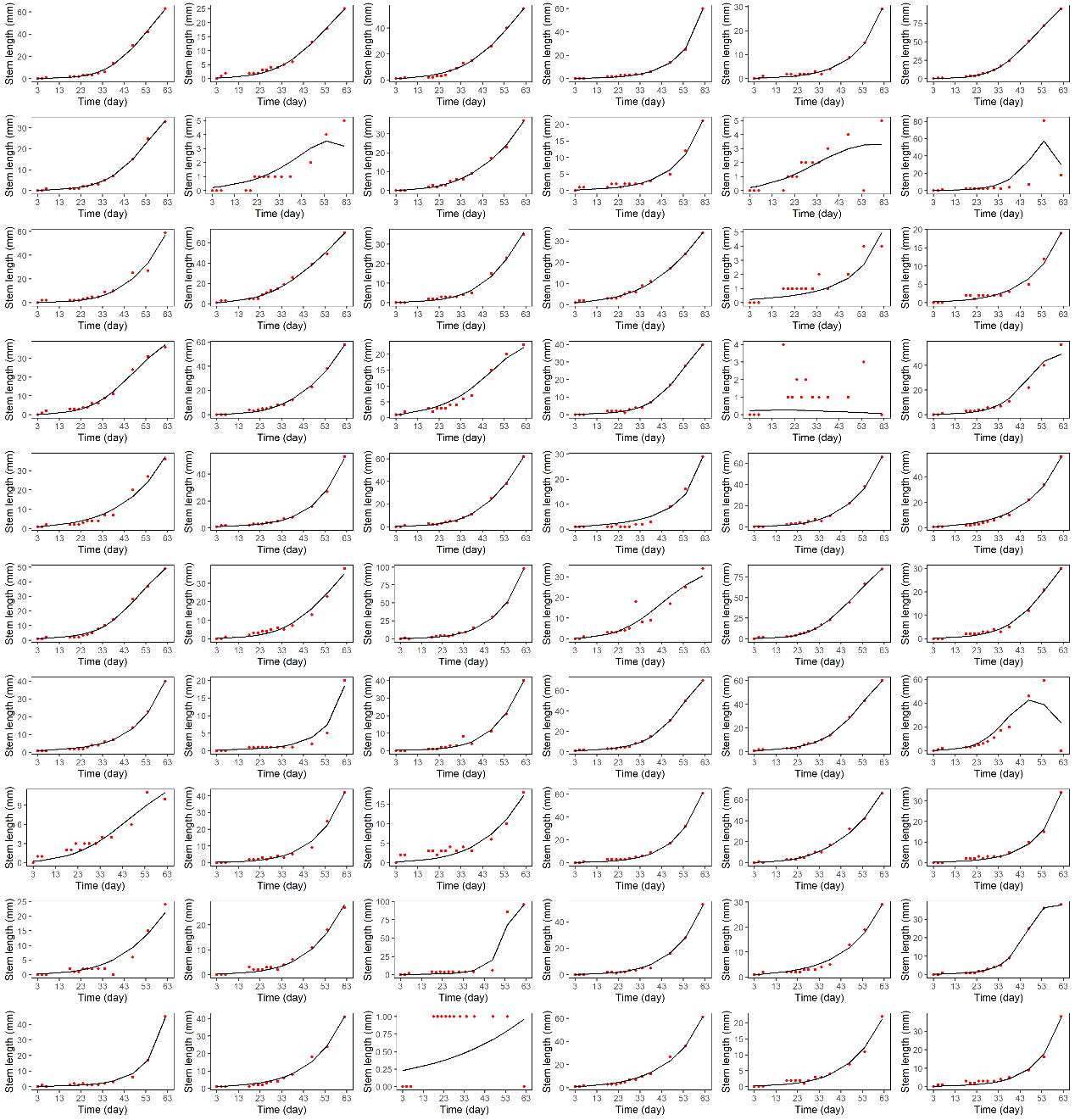


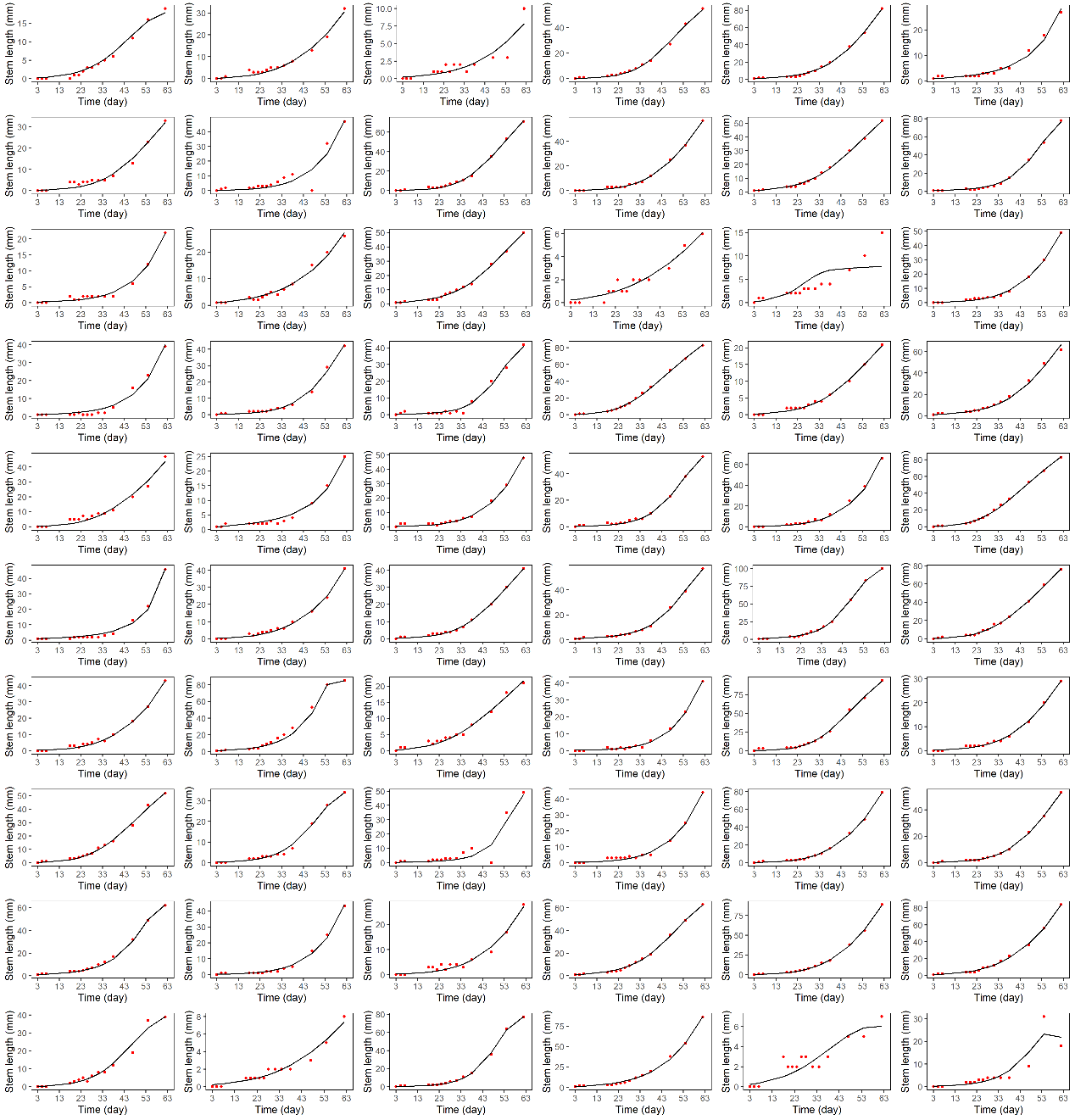


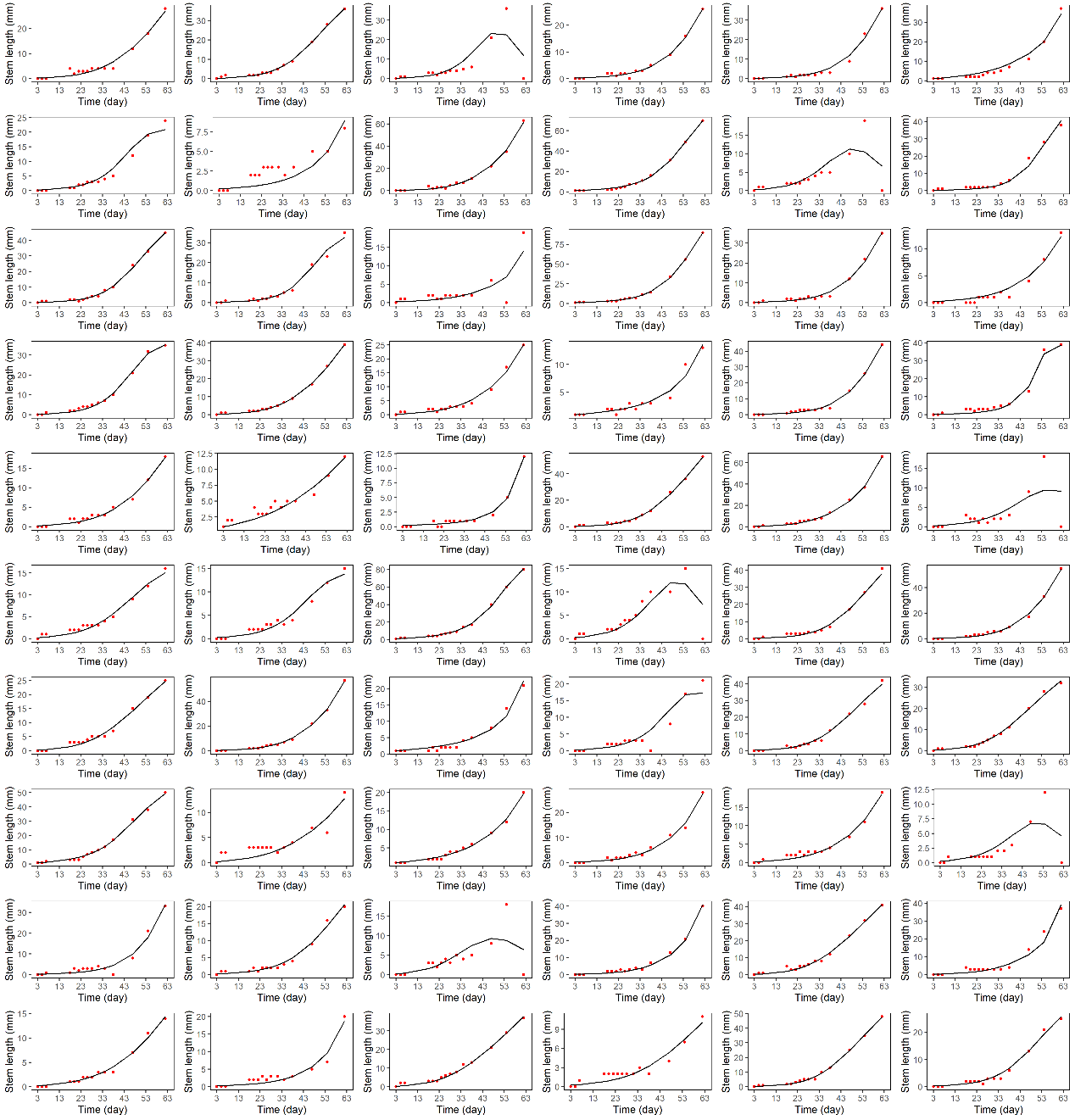


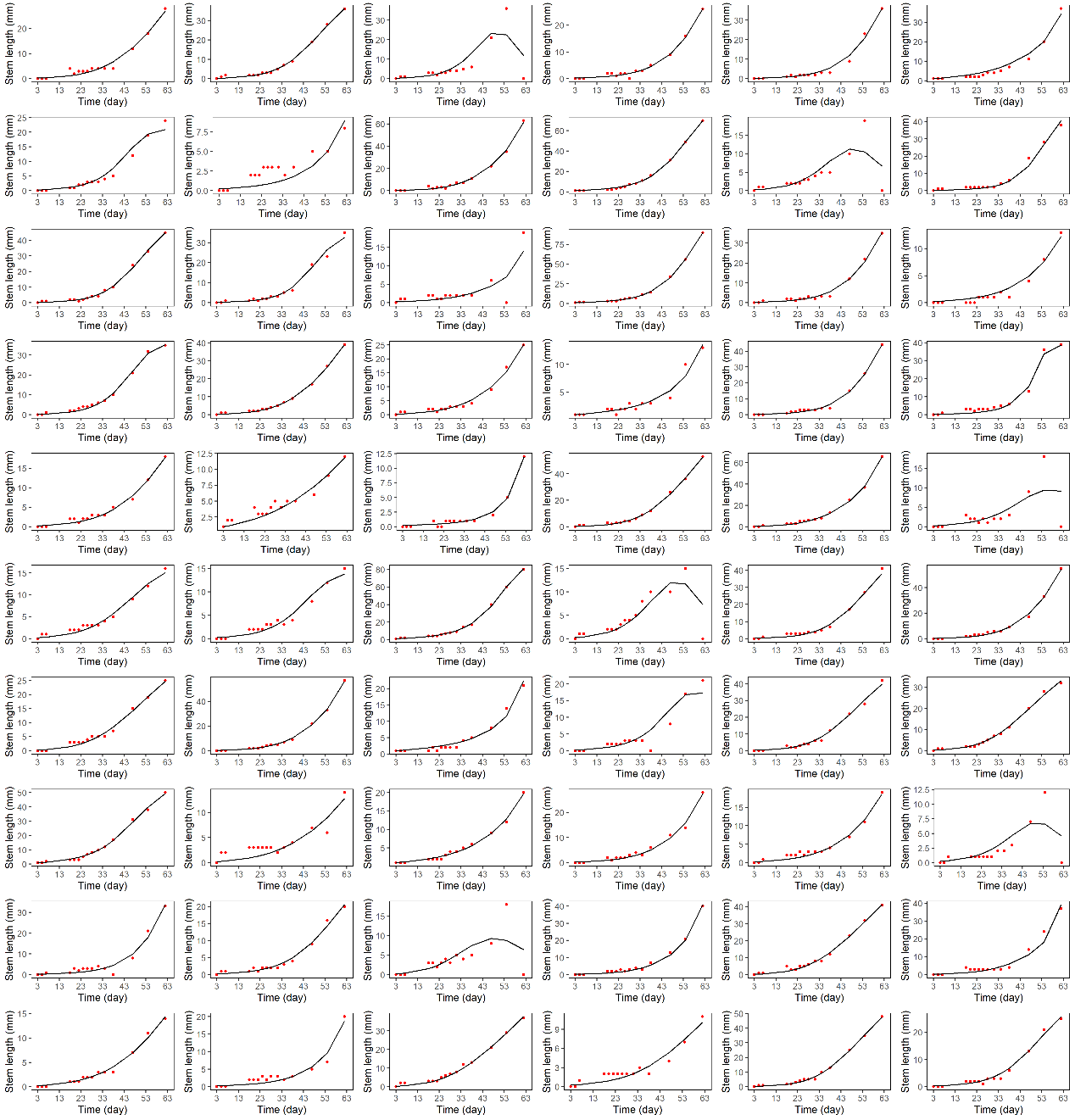


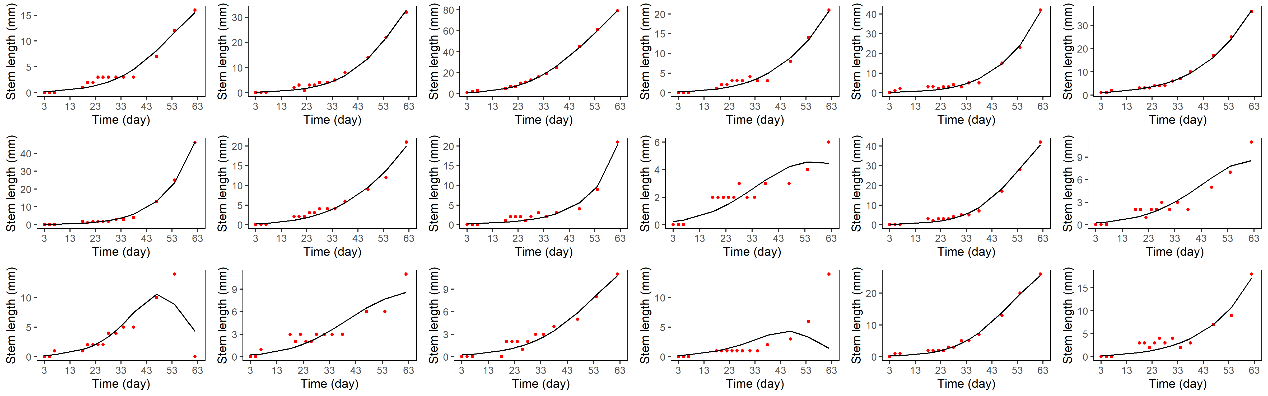


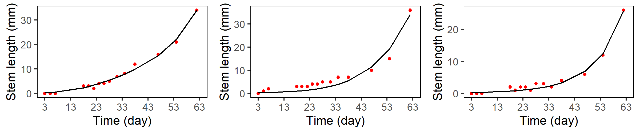


**Supplementary Figure 2**

**
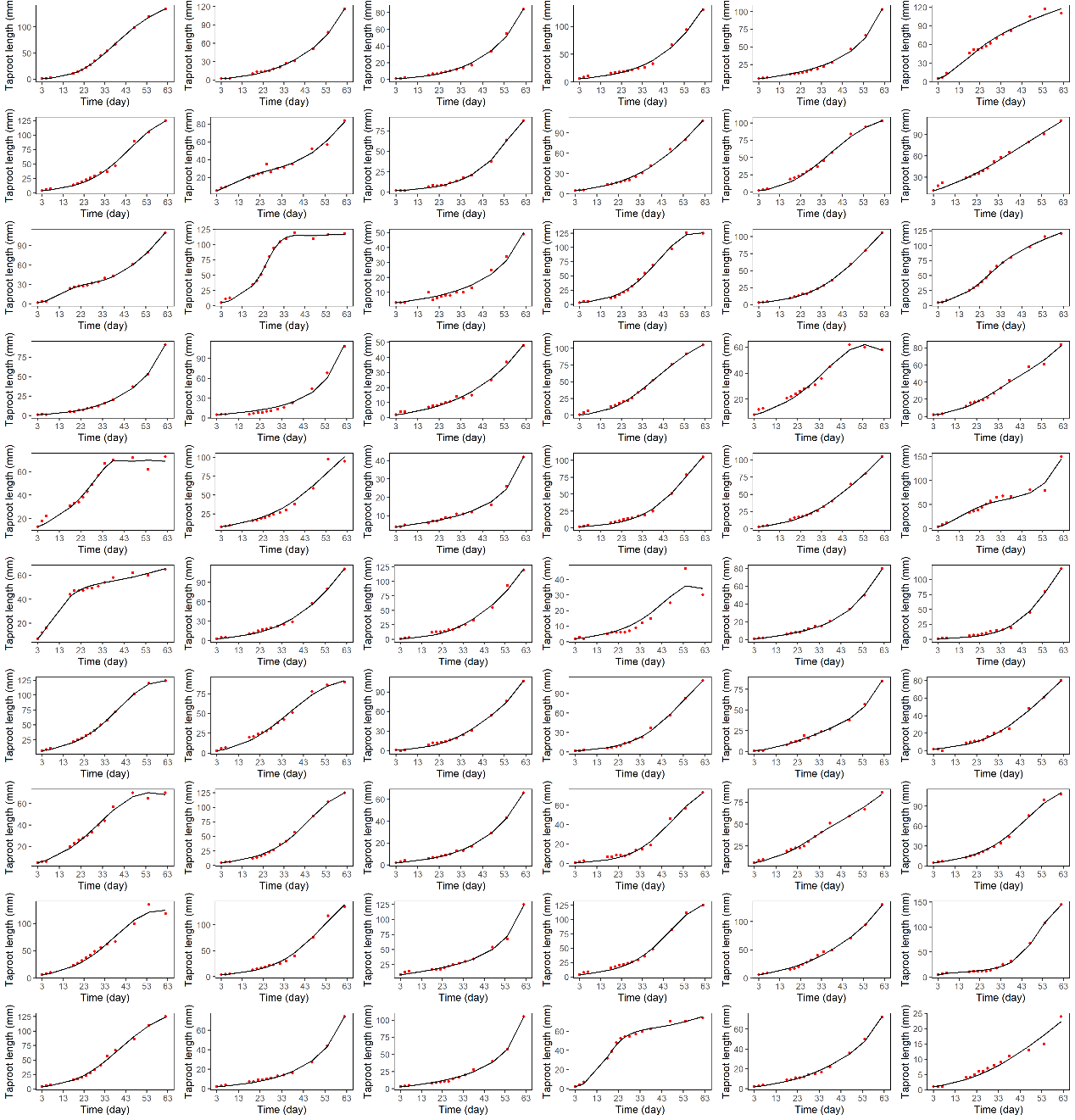
**

**
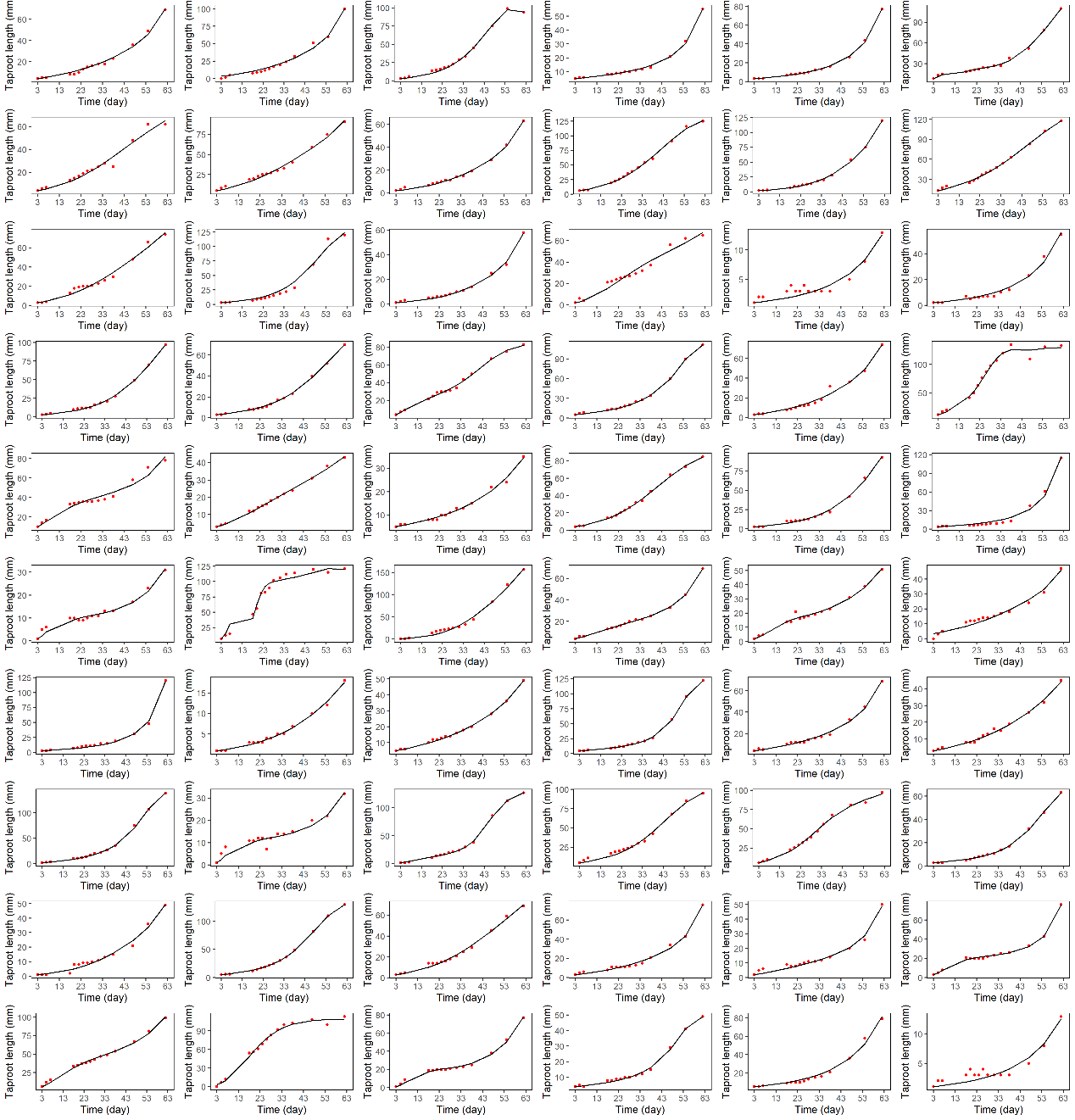
**

**
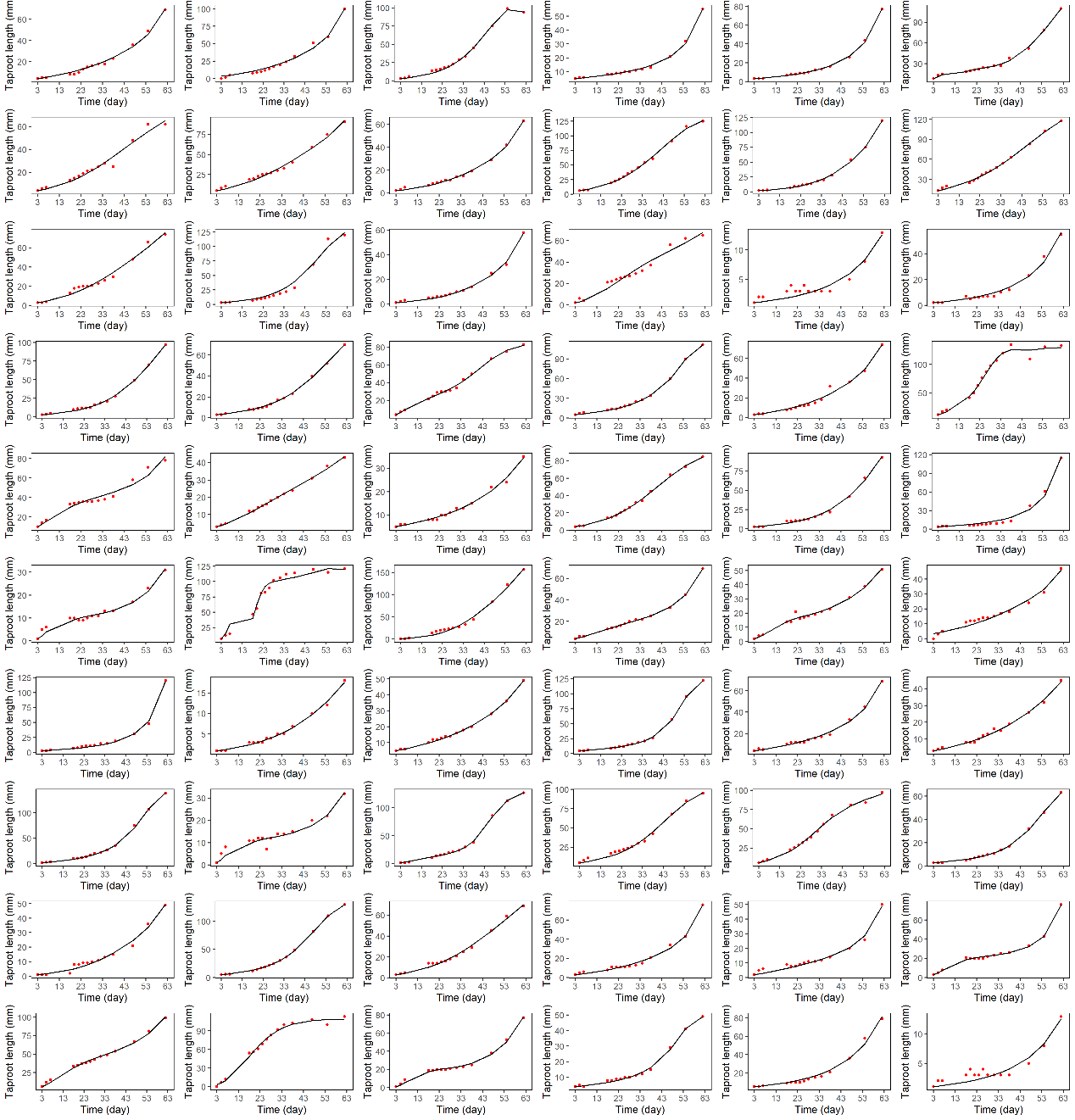
**

**
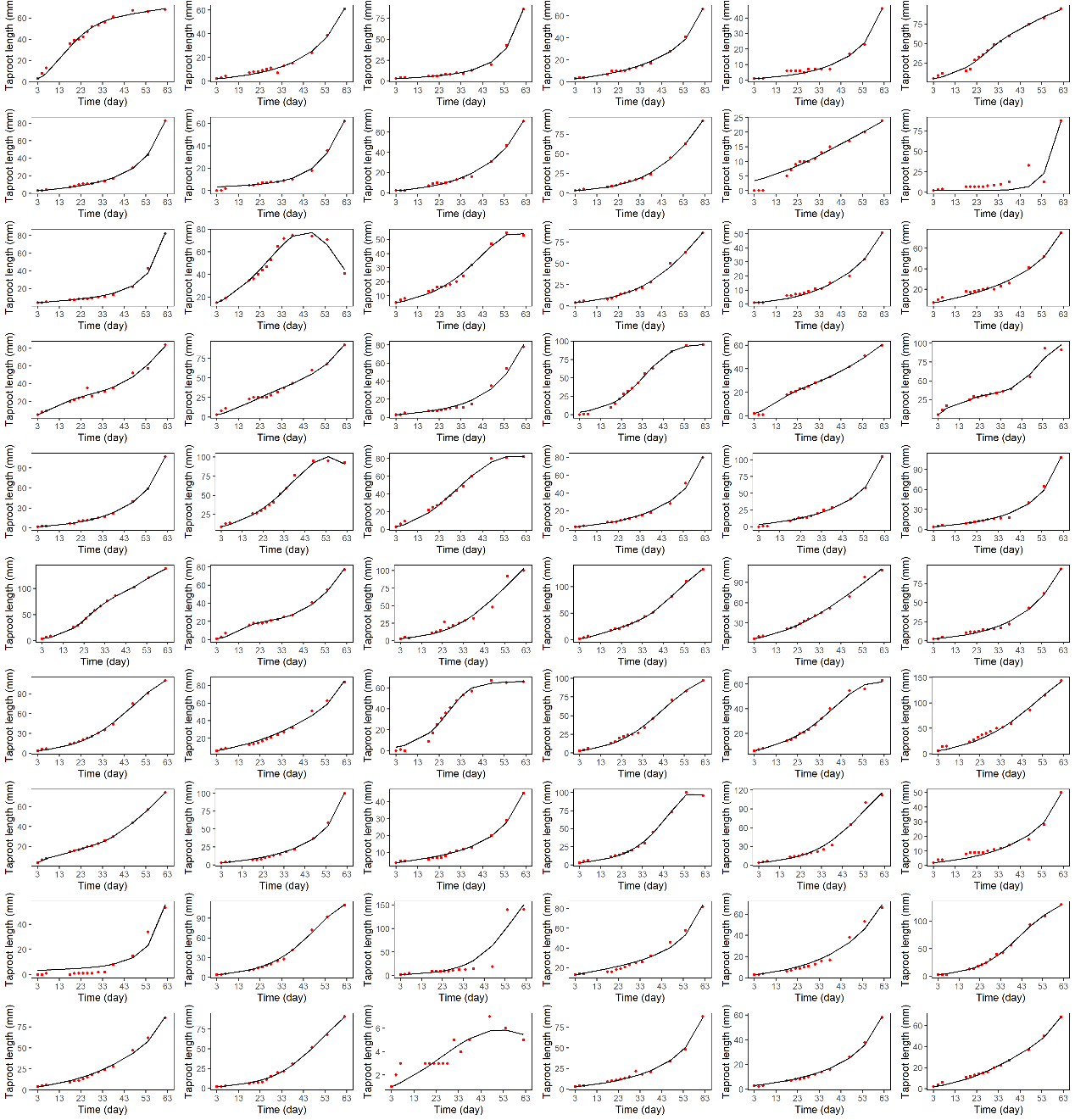
**

**
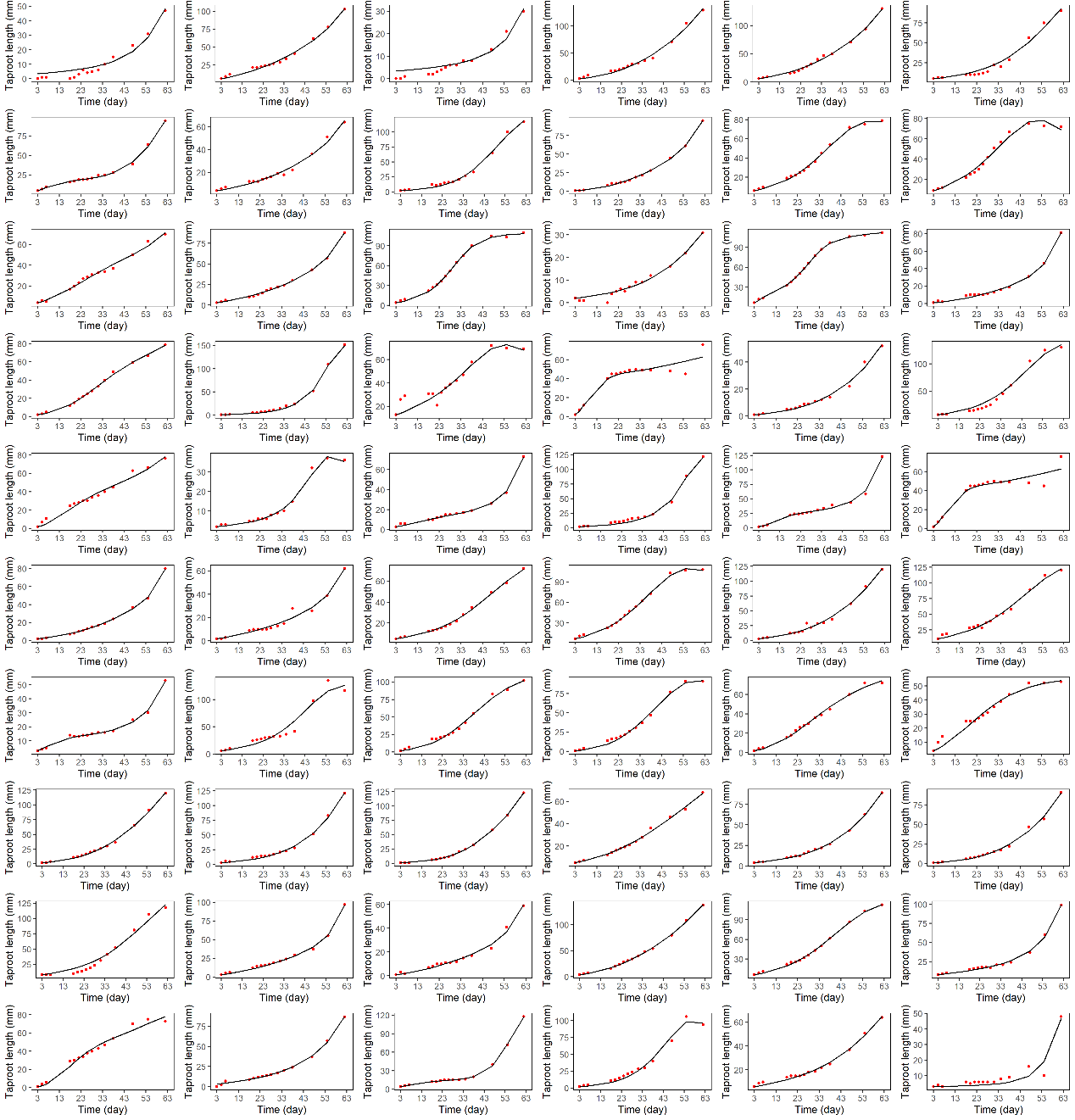
**

**
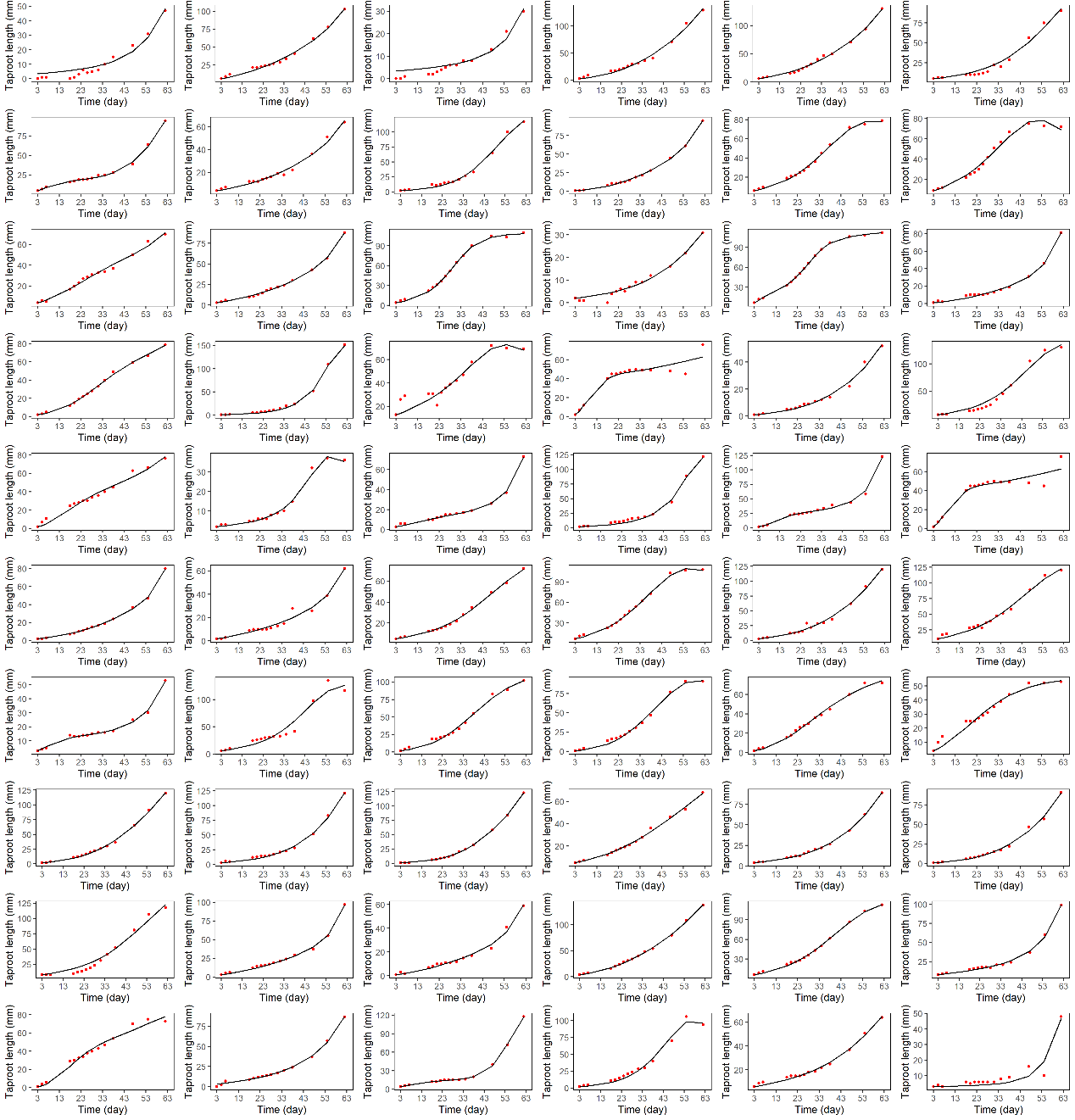
**

**
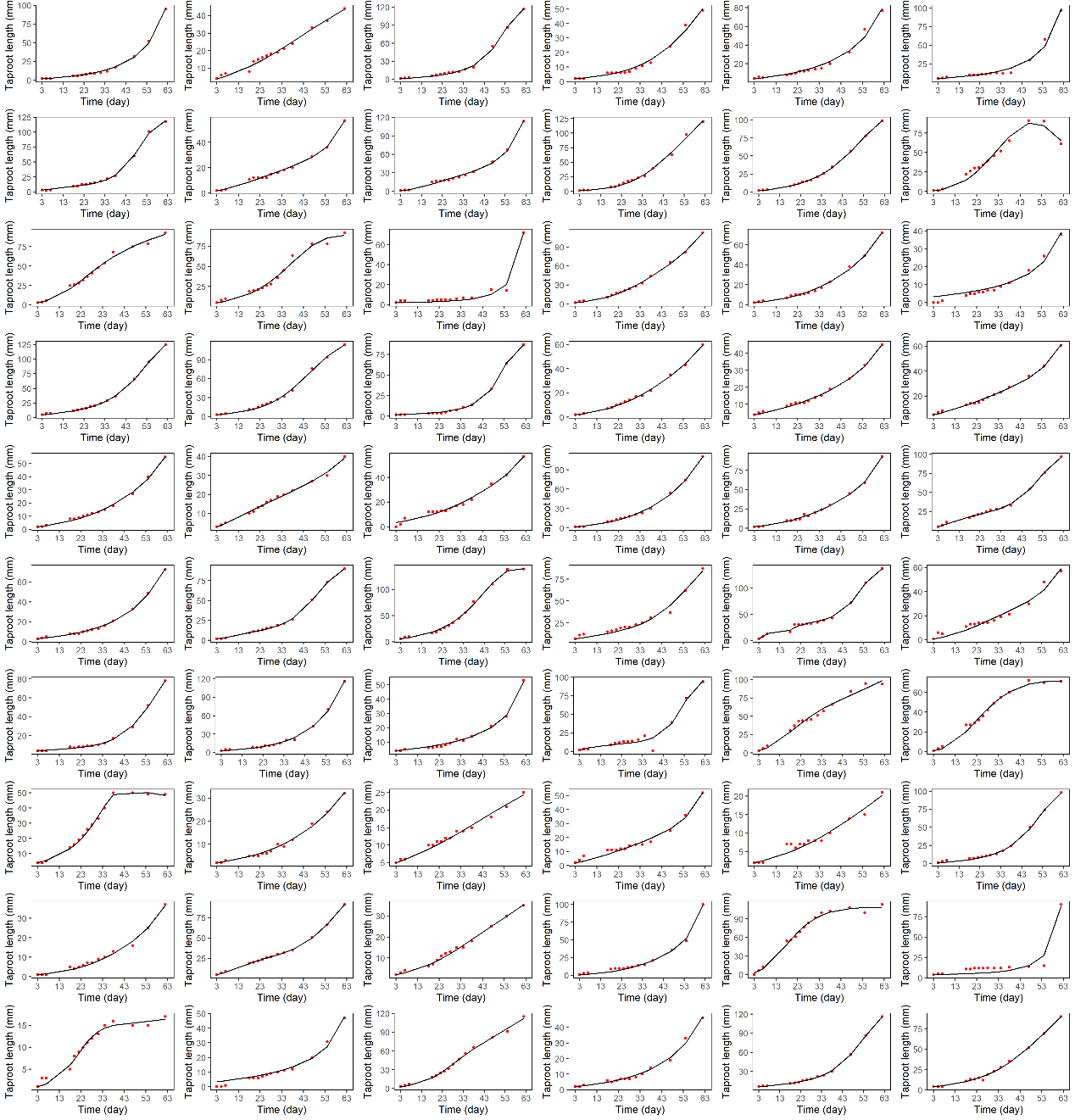
**

**
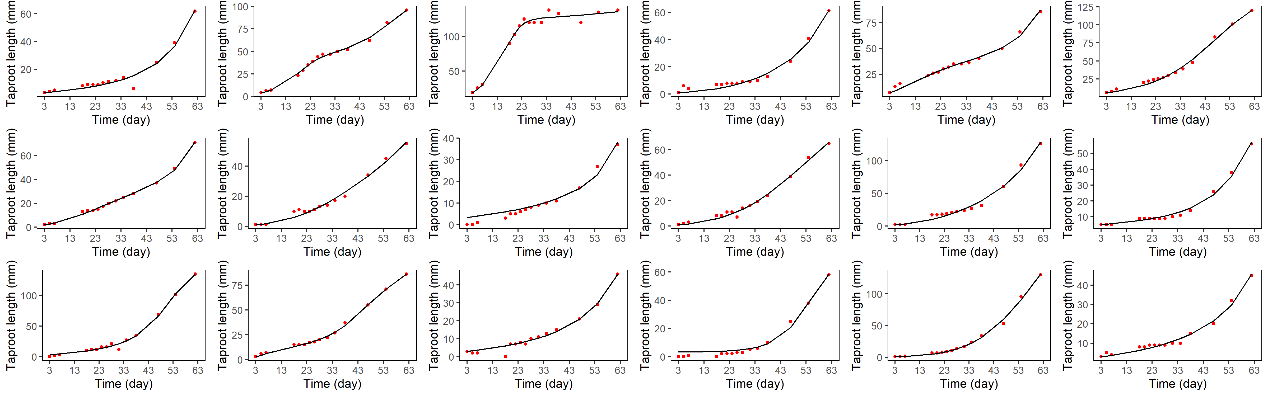
**

**
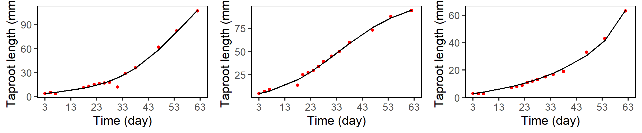
**

**Supplementary Figure 3**

**
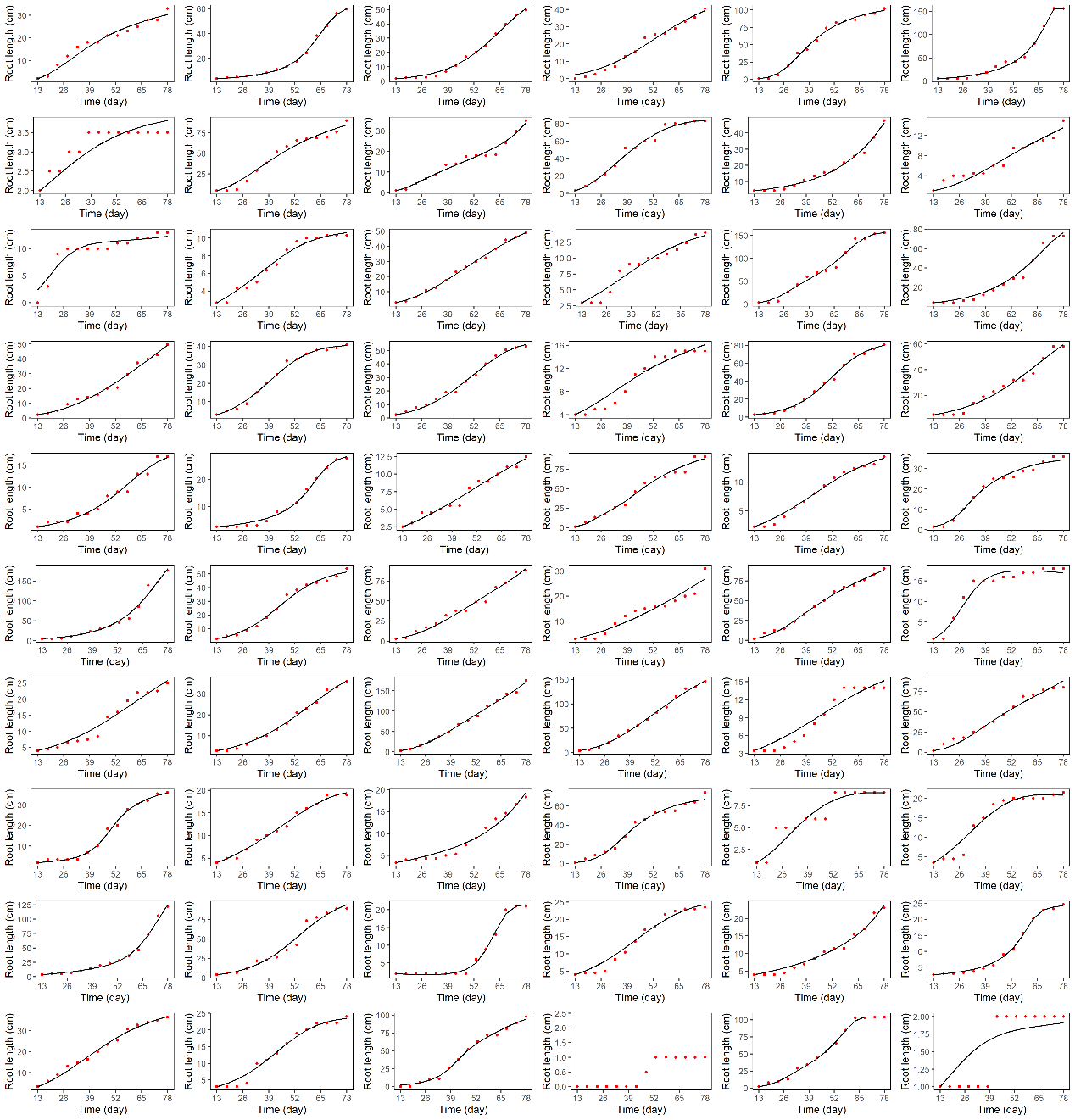
**

**
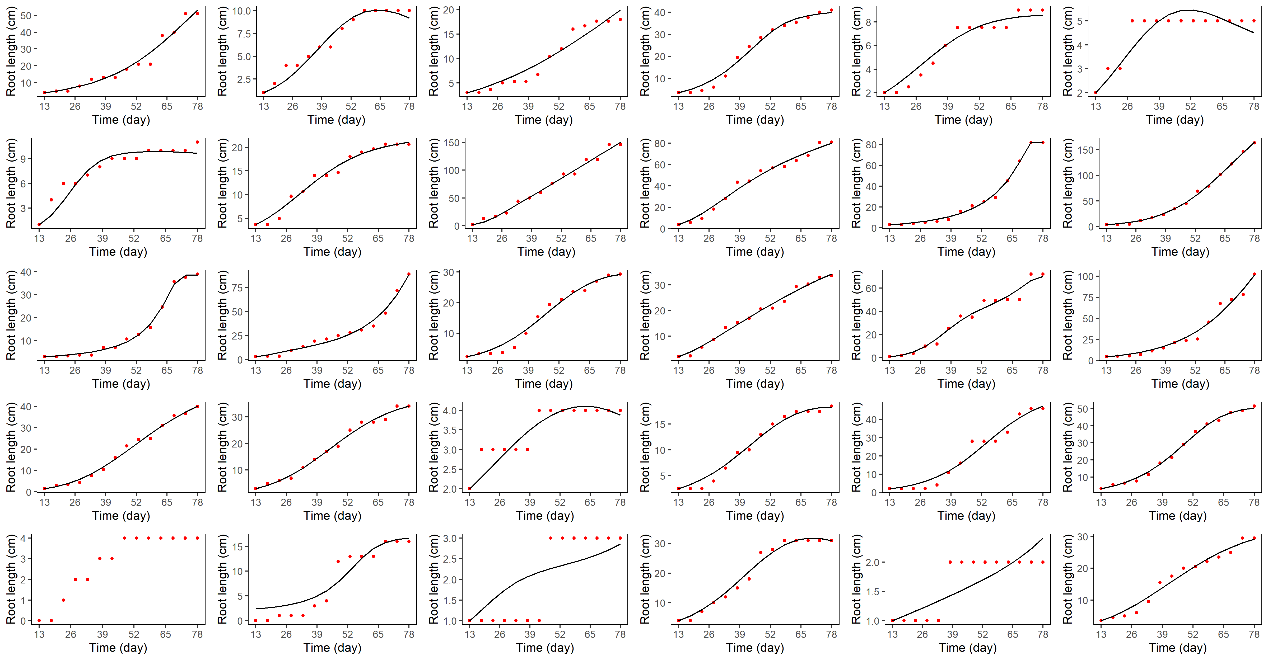
**

**
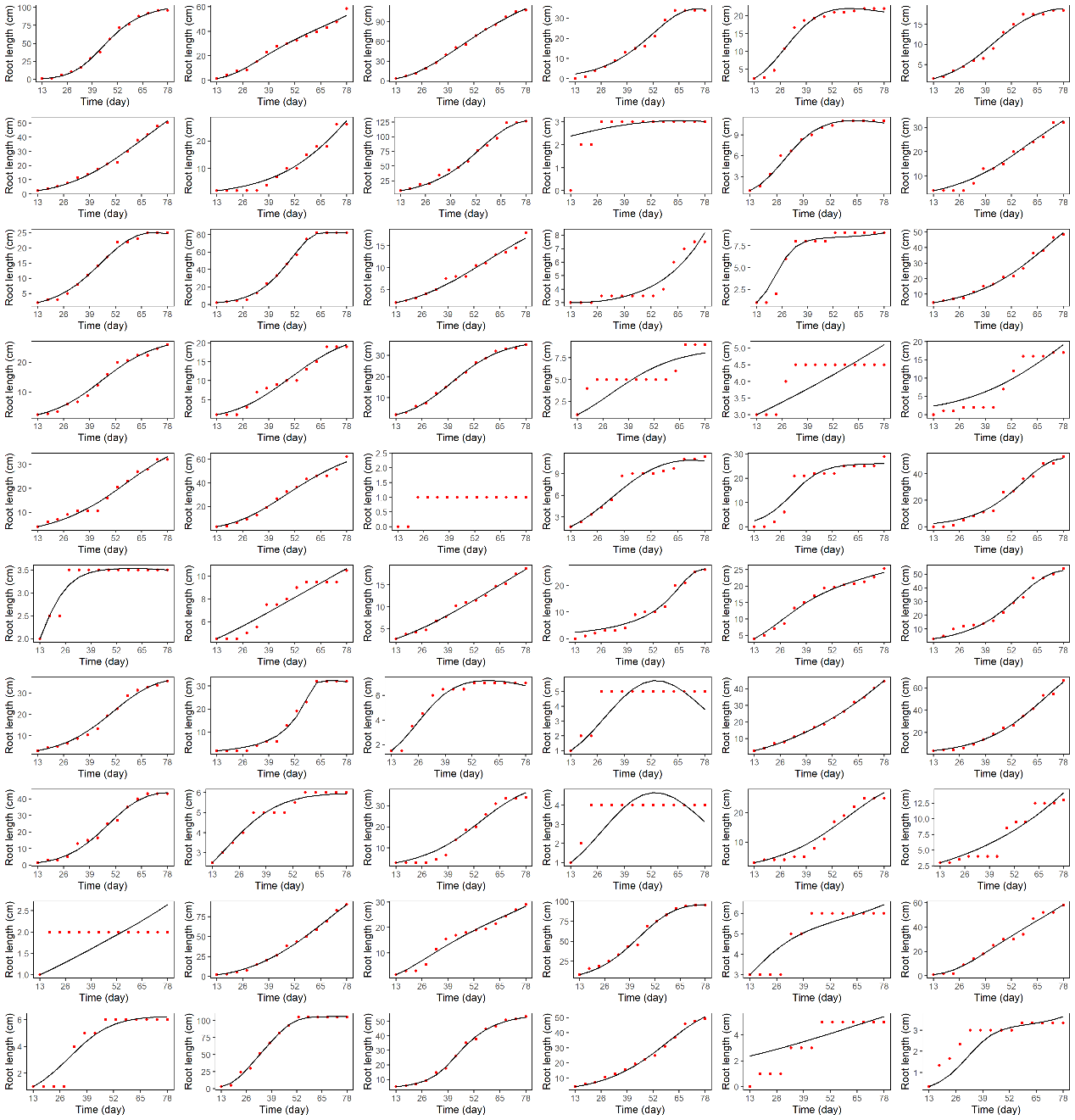
**

**
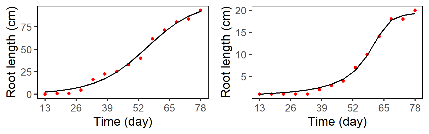
**

**Supplementary Figure 4**

**
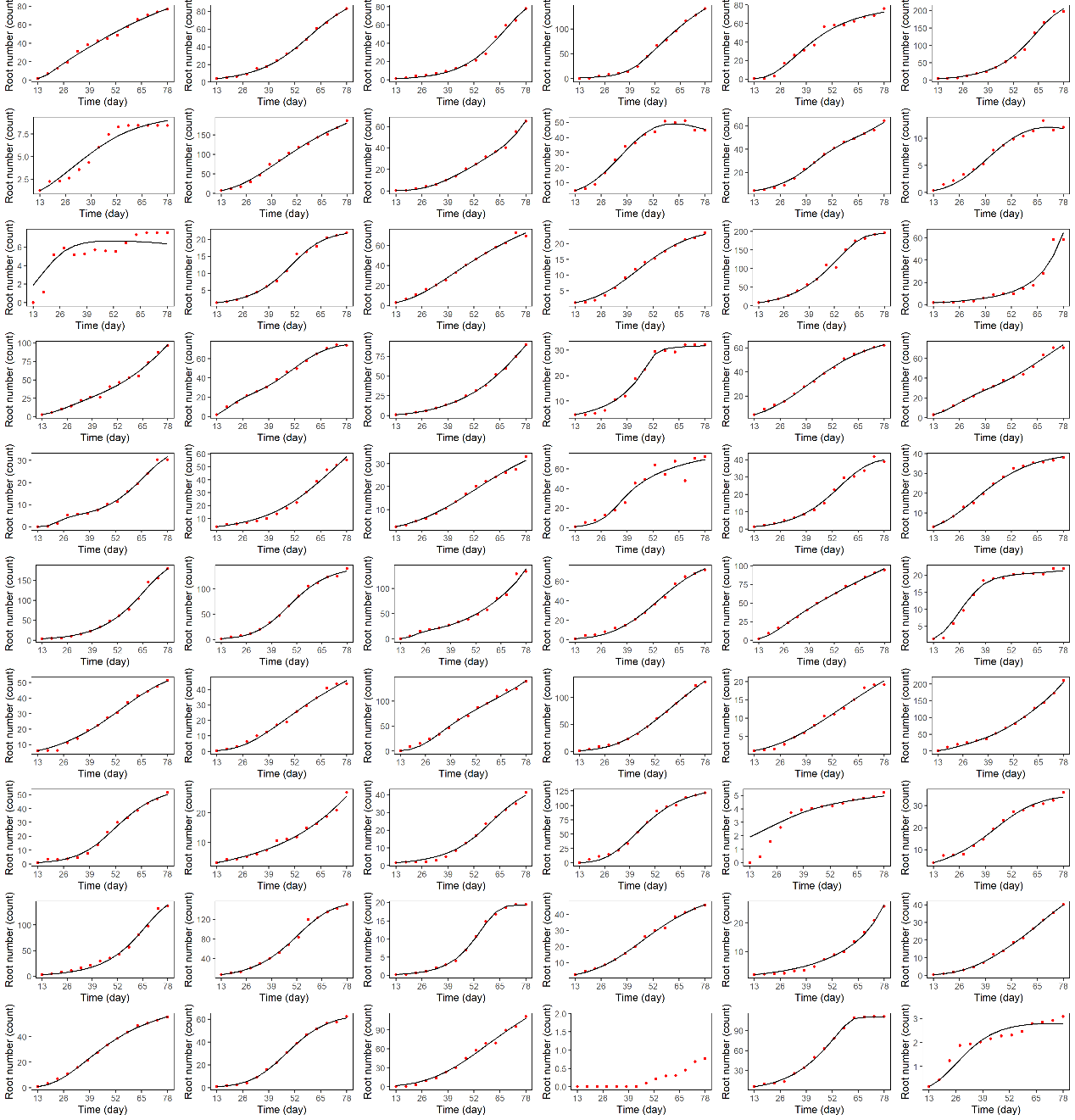
**

**
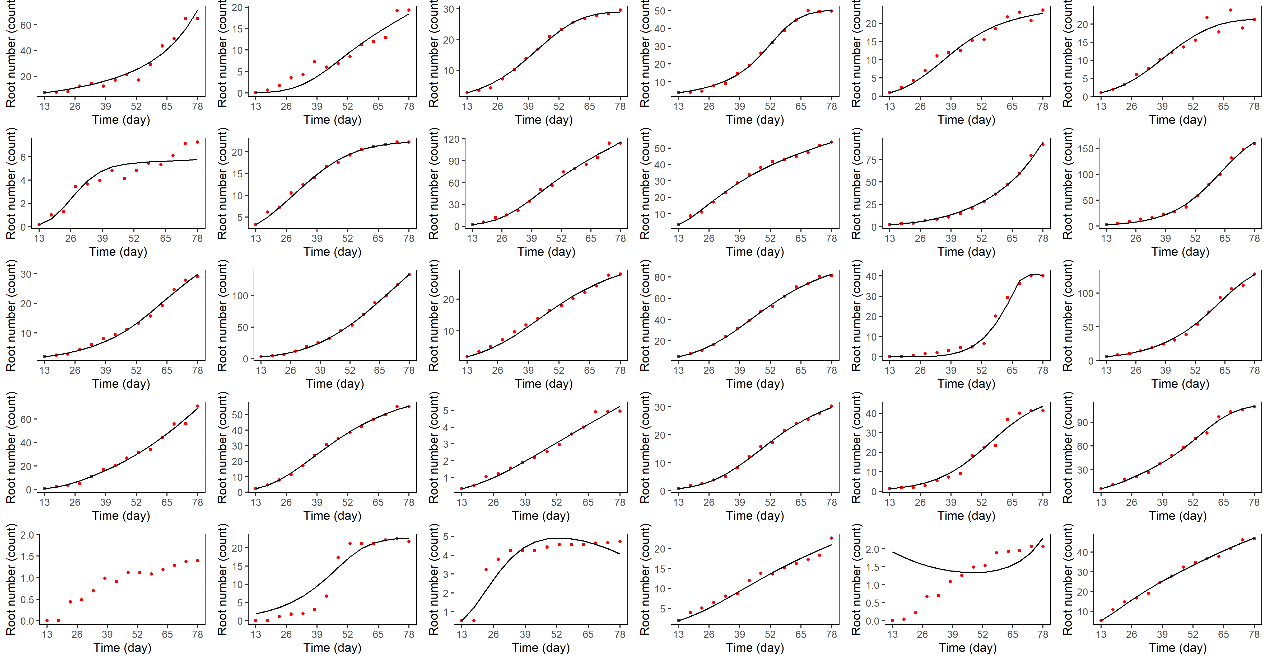
**

**
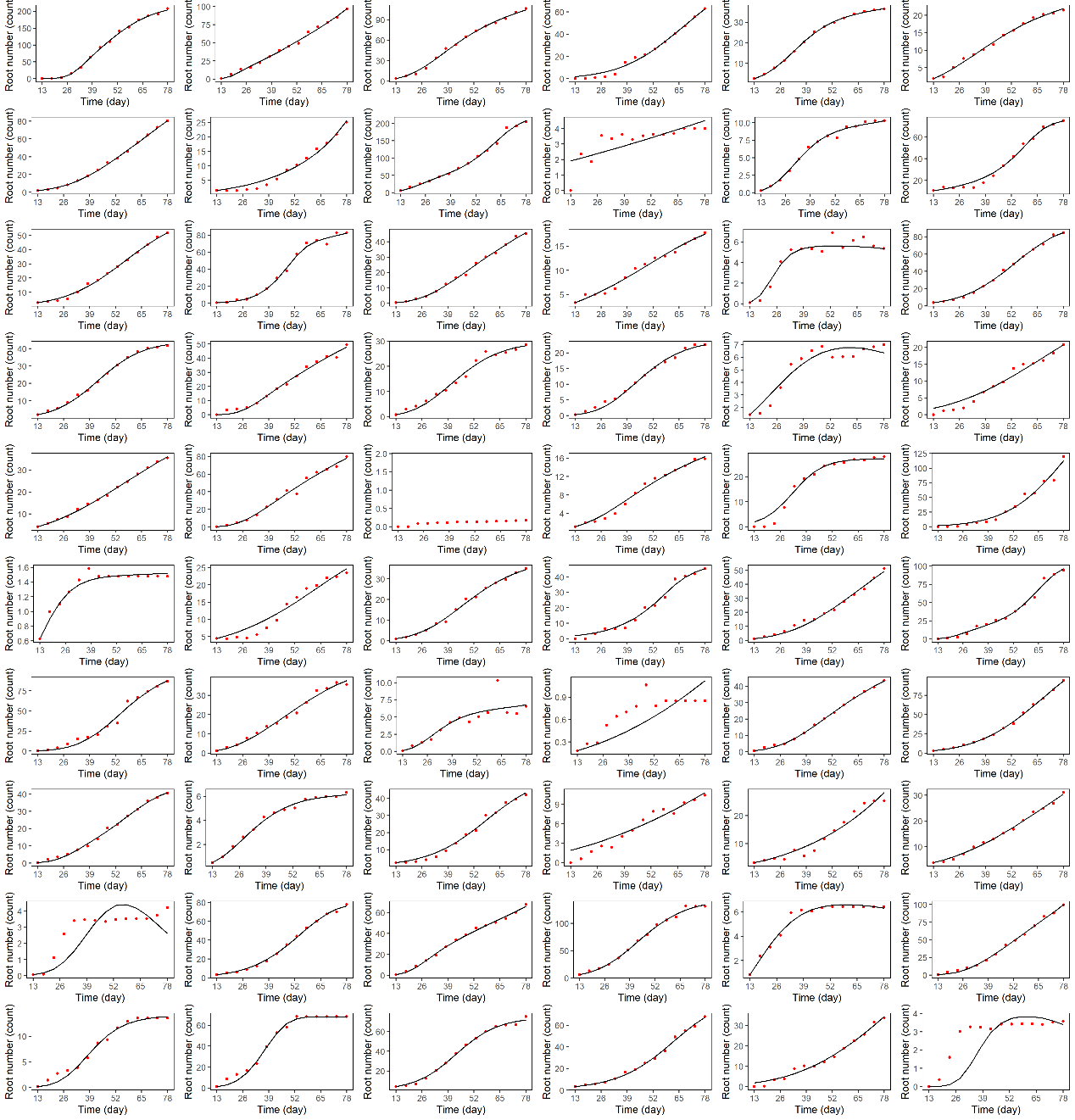
**

**
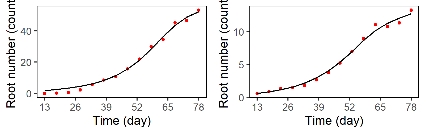
**

**Supplementary Figure 5**

**
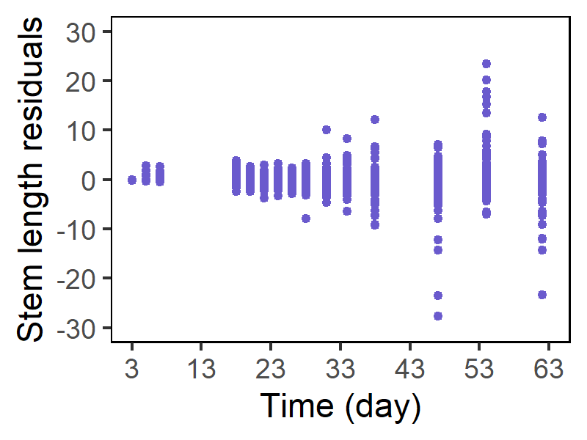

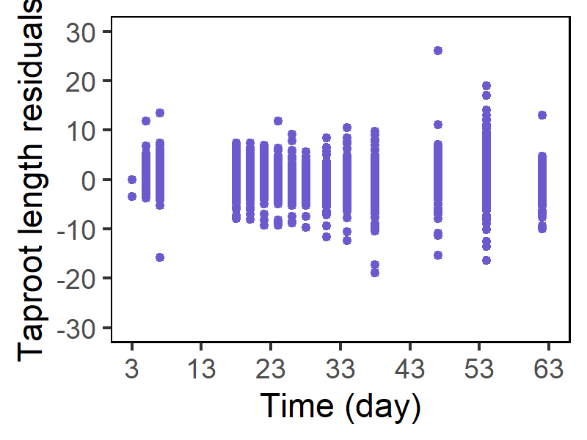
**

**
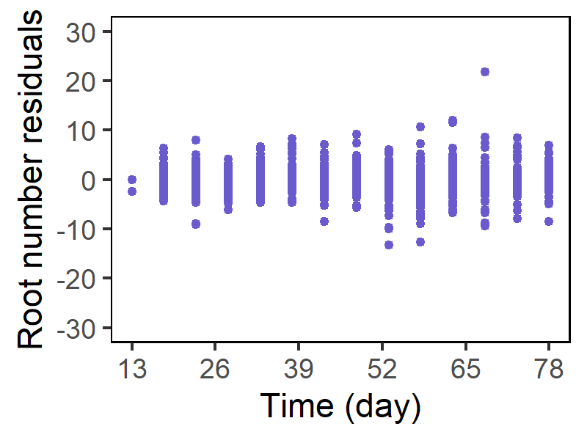

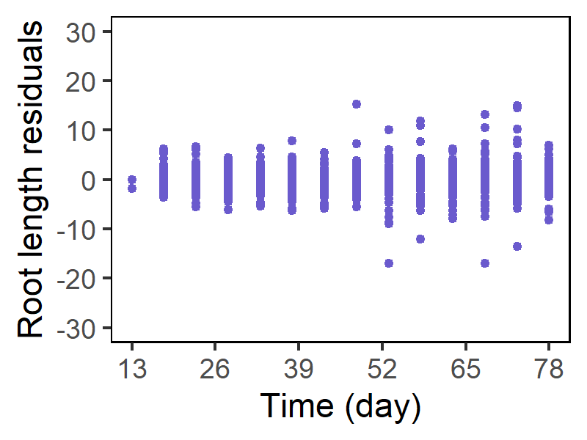
**
